# A Model-Driven Co-Design Framework for Fusing Control and Scheduling Viewpoints

**DOI:** 10.3390/s18020628

**Published:** 2018-02-20

**Authors:** Sakthivel Manikandan Sundharam, Nicolas Navet, Sebastian Altmeyer, Lionel Havet

**Affiliations:** 1 Laboratory of Advanced Software Systems (LASSY), CSC Research Unit, University of Luxembourg, Maison du Nombre, L-4364 Esch-sur-Alzette, Luxembourg; nicolas.navet@uni.lu; 2 CSA Group, University of Amsterdam, 1098XH Amsterdam, The Netherlands; altmeyer@uva.nl; 3 RealTime-at-Work (RTaW), 4 Rue Piroux, 54000 Nancy, France; lionel.havet@realtimeatwork.com

**Keywords:** model-driven engineering, control software, timing tolerance contract, controller model, schedulability, stability, input jitters, varying execution-times, output jitters, input-to-output delay, co-simulation, real-time scheduling, control system performance

## Abstract

Model-Driven Engineering (MDE) is widely applied in the industry to develop new software functions and integrate them into the existing run-time environment of a Cyber-Physical System (CPS). The design of a software component involves designers from various viewpoints such as control theory, software engineering, safety, etc. In practice, while a designer from one discipline focuses on the core aspects of his field (for instance, a control engineer concentrates on designing a stable controller), he neglects or considers less importantly the other engineering aspects (for instance, real-time software engineering or energy efficiency). This may cause some of the functional and non-functional requirements not to be met satisfactorily. In this work, we present a co-design framework based on timing tolerance contract to address such design gaps between control and real-time software engineering. The framework consists of three steps: controller design, verified by jitter margin analysis along with co-simulation, software design verified by a novel schedulability analysis, and the run-time verification by monitoring the execution of the models on target. This framework builds on CPAL (Cyber-Physical Action Language), an MDE design environment based on model-interpretation, which enforces a timing-realistic behavior in simulation through timing and scheduling annotations. The application of our framework is exemplified in the design of an automotive cruise control system.

## 1. Introduction

Control theory and software engineering are two disciplines involved in the development of control software. Traditionally, control engineers design the controller model without considering the computing platform constraints and specifications. The converse applies to software engineering, where control performance is not considered during software design. The control engineering and the software engineering are two different worlds with different objectives in mind. Consequently, the complete set of functional and non-functional requirements of the control software are usually not elicited at the control design stage. Hence, as discussed in [1], substantial design-gaps may exist during the design of a control software.

The control software executes on an Electronic Control Units (ECU) interfaced with various sensors and actuators. The continuous-time signals are periodically sampled; each sampled set of data is then processed by real-time control functions. Control theory typically assumes deterministic and periodic sampling. However in practice, for instance, due to preemptions and varying task execution times, there exists a varying delay between sensing and actuation, which is called input-to-output delay or sensing-to-actuation delay. A control designer typically assumes this input-to-output delay to be zero or constant which is an unrealistic assumption. The input-to-output delay depends on the time at which sensing and actuation takes place. Sensing time may also vary over time typically due to the interference of higher priority tasks, and the variability of sensing times is called *input jitter*. There are also jitters in the actuation times, called *output jitters* caused by varying execution times and preemptions. These jitters directly impact the quality of control functions, and, in the worst-case, they might jeopardize the safety of the system. Hence, it is important to consider these delays during the design phase of the control software. This work addresses the case where the input data acquisition is done locally on one node. It can be extended like in TrueTime [2] to cover the case of networked control systems, including "Industrial Internet of Things" (IIoT) applications, where data are transmitted over a network, which would increase the input jitters, as well as the input-to-output delays.

### 1.1. State-of-the-Art

A survey of tools and methods developed to address this problem is presented in [3]. Most of the techniques discussed in this survey are based on co-design approaches. Directly relevant to our work are TrueTime [2] and T-Res [4] which are simulation tools that can consider how the timing behavior of the implementation affects the performance of the control. Both approaches use Simulink for control design and timing extension toolboxes to include computing aspects, foremost the effects of task scheduling on the control performance. In a recent study [5], we discussed our co-design and simulation environment and compared it with these state-of-the-art tools. Our co-design technique mainly differs from these approaches by allowing the control model to be directly executed (by an interpreter engine) on the target hardware, without changing a single line of code. The benefits are reduced development time and avoidance of distortions (i.e., semantic gaps) between the simulated and executed control programs. On the other hand, TrueTime and T-Res are essentially simulation environments that involve a step of model-to-code transformation (typically code generation), which may risk widening the semantic gap between model and executable code, requiring additional development effort. Generally speaking, the existing co-design simulation techniques are mainly concerned with enabling the study of the effect of timing variabilities on control performance, rather than addressing the design gaps between control and software viewpoints. Other works [1,6] present co-engineering techniques where the initial controller is integrated in a virtual ECU. The behavior of the controller is then assessed through timing analysis tools whose results are injected into the controller model. This approach shares similarities with ours but it relies on expensive and proprietary timing analysis tools and remains at the model level (i.e., implementation is abstracted).

### 1.2. Contributions

In this paper, we propose a framework that supports our co-design modeling environment for both controller and control software development. The framework provides schedulability and control performance analysis along with simulation capabilities. We underpin the proposed framework with the help of timing contracts introduced in [7] which are sets of timing characteristics that ensure the targeted control performance. The timing contract can be a crucial concept in component-based design because it drives and synergizes the design thinking of the stakeholders from different viewpoints. We use the timing contract as a candidate to bridge the control software design-gaps. During the application of a timing contract, we observe a vertical type contract [8] in our proposed framework as the timing contract is applied between two phases of the Software Development Life Cycle (SDLC), in this case between controller design and software development.

The co-design framework presented in this work encompasses three steps of the development cycle: (i) controller design; (ii) software scheduling and execution platform configuration; and (iii) run-time monitoring. Firstly, we discuss scheduling and stability viewpoint analyses supporting the proposed co-design and simulation environment. We rely on our timing-aware model-driven environment called Cyber-Physical Action Language (CPAL) for co-design in Simulink. We then present the CPAL constructs and timing annotations, central to our approach, which enable us to reproduce the timing irregularities of interest, such as jitters and varying input-to-output delays. CPAL provides the timing dimension to the controller design, which acts on the plant model in Simulink. We provide the CPAL execution platform for Simulink as open access for experimentation. Along with existing jitter analysis tools, the proposed co-design platform helps designing stability guaranteed controller models by integrating the target-platform timing behavior. Furthermore, it provides software engineering with the control information needed to bound the space of feasible software design solutions. The stability verification itself is done with the help of the jitter margin concept and the co-simulation of CPAL execution in the Simulink environment.

The second contribution is the verification of the timing tolerance contract assumptions made during controller design. The verification is specifically useful when a new control function is integrated into an existing stable and functioning ECU. How can we analytically validate whether the system maintains the desired performance (stable and schedulable) after integration? To this end, we propose a novel schedulability analysis for a certain class of task and execution models in real-time scheduling. To assign a realistic execution time to the controller task, we estimate the Worst-Case Execution Time (WCET) beforehand using measurements of the model running on the target hardware.

The third and last contribution is the proposed run-time verification methodology. During model on target execution, we check whether the newly integrated controller function stays within the stability margin. For this, we take advantage of CPAL introspection features to monitor the execution characteristics of a controller model at run-time. More specifically, we introspect whether the jitters and input output latencies are within the margin guaranteeing the stability and schedulability objectives.

### 1.3. Structure

This paper is structured as follows. In Section 2, we explain the system model and the steps involved in the framework for fusing control and scheduling viewpoints. Section 3 presents the proposed co-modeling and simulation environment as well as jitter analysis tools and methods. In Section 4, we explain the verification of timing tolerance assumptions using WCET measurements and the schedulability analysis. In Section 5, we evaluate the framework using the example of a cruise control system. In the same section, we discuss the stability verification using the jitter margin concept and the CPAL co-simulation in Simulink. The section also details the scheduling configuration and run-time introspection features. Section 6 provides the related work. Section 7 concludes the paper.

## 2. Framework for Fusing Control and Scheduling Viewpoints

System designers in the industry are typically highly knowledgeable in their own fields (control systems, software engineering, scheduling, etc.) but contracts among design teams are not necessarily well established and communicated among the stakeholders. Our objective is to define a structured framework, with clear interfaces, which can be agreed upon and followed by all. The framework proposed in this section highlights the issues faced at each step of the design and we propose possible solutions. Our framework may not be suitable for all industrial settings, but it addresses the gap between control models and their implementation, and can serve as a basis for context-specific design frameworks.

### 2.1. System Model

We propose an integrated framework which combines the tools and methods necessary to design a model of the system. Table 1 provides a quick reference for the notations used in this paper. The system is comprised of a controller model, a plant model and platform model. Plant *P* is modeled by a continuous-time system of equations

(1) $$\begin{array}{cc} \dot{x} & =Ax+Bu,\\ y & =Kx, \end{array}$$

where *x* is the plant state and *u* is the control signal. The plant output *y* is sampled periodically with some delays at discrete time instants. The control signal is updated periodically with some delays at discrete time instants, (i.e., actuation also happens with some delay). Quantities *A*, *B*, *K* are constants. The controller model is comprised of a task set Γ of *n* periodic tasks $\left\{T_{1},\ldots T_{n}\right\}$ executing on a single processor.

Each controller task $T_{i}$ is represented by a tuple $T_{i}:\left(O_{i},C_{i},h_{i},D_{i}\right),$ where $O_{i}$ is the task’s release offset, $C_{i}$ the Worst-Case Execution Time (WCET), $h_{i}$ the task’s period and $D_{i}$ the deadline. $R_{i}^{w}$ and $R_{i}^{b}$ are the worst and best-case response times. The task instances, also referred to as jobs, are scheduled non preemptively in order of their arrival. Each controller task is assumed to have three activities in the order sensing, computation and actuation. Sensing is the first activity which reads the data from a sensor. The computation also known as *control law execution* is the second activity. The actuation is the last activity which writes the data to physical devices.

The variability in the times at which the control software reads and writes the input and output data is called jitter. Jitters have a major impact on the performance of some control systems. To formally define the jitters that must be respected by an execution platform, the authors in [7] introduce four timing contracts namely Zero Execution Time (ZET), Bounded Execution Time (BET), Logical Execution Time (LET) and Timing Tolerance (TOL) contract. In this work, we consider the latter contract which is more general than ZET and BET, and does not imply strong implementation constraints like LET [9]. A Timing Tolerance TOL contract implies that the following conditions hold:

(2) $$\begin{array}{cc} t_{k}^{s}\; & \epsilon \;[k.h,k.h+J^{h}],\\ t_{k}^{a}\; & \epsilon \;[t_{k}^{s}+\tau -J^{\tau },t_{k}^{s}+\tau +J^{\tau }], \end{array}$$

where $J^{h}$ is the tolerable input jitter. τ is the tolerable input-to-output delay also known as tolerable Sensing-to-Actuation delay (StA delay). The nominal input-to-output delay L is a minimum delay experienced between input to output. $J^{\tau }$ is the tolerable output jitter. The tolerances $J^{h}$ and $J^{\tau }$ are also referred to as margins, namely input jitter margin and output jitter margin.

### 2.2. Framework Steps

**Figure 1:**
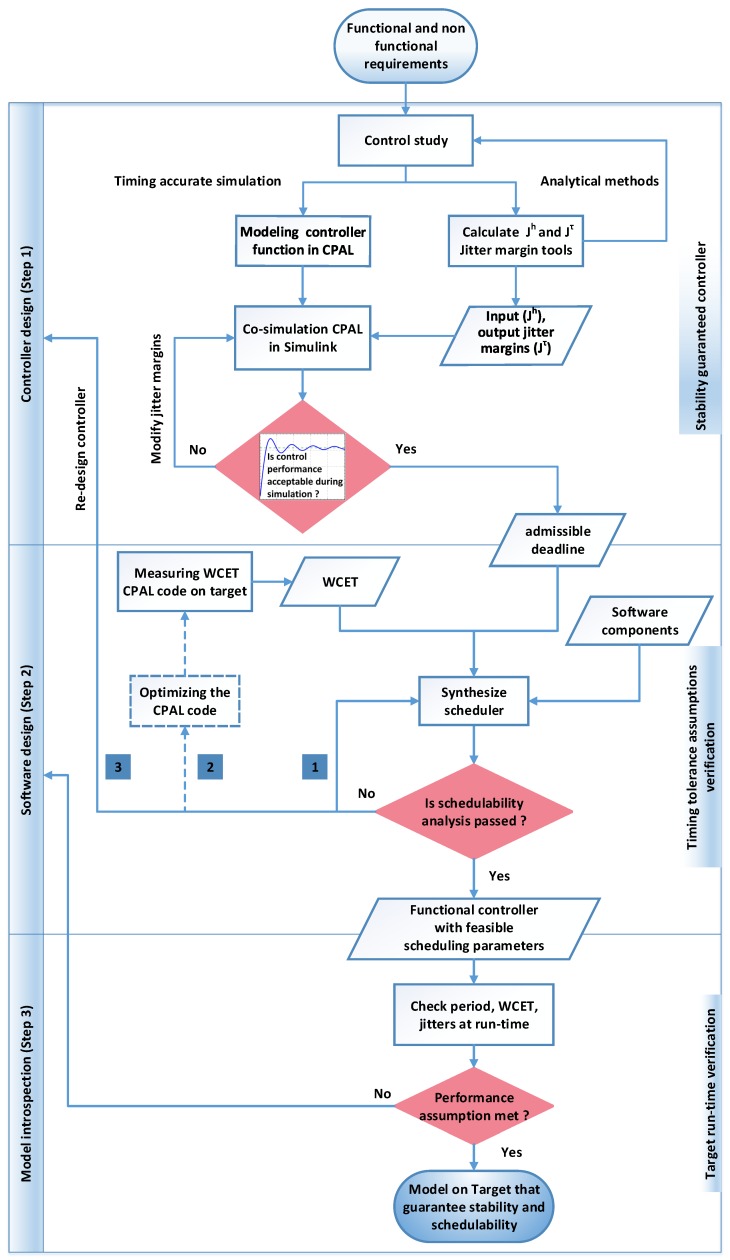
Illustration of framework flow for fusing control and scheduling viewpoints. The dashed part in the software design step is out-of-scope of this paper.

To bridge the control-computing gap, we propose a framework that fuses the control and scheduling viewpoints in the context of model-based system design. Figure 1 shows the overall step-by-step flow of the framework.

#### Step 1: Controller Design

Based on the functional and non-functional requirements, the first stage of the framework is the control study, that determines the control equations that will potentially allow the system to achieve the required control performances. This control study relies on the designer’s expertise with the help of control-system simulators like MATLAB/Simulink, which include the plant model. We note that at this stage the timing issues are not considered, and in particular the implementation delays are ignored, which may require to revisit the choice of the control law later in the design flow.

The next stage is to model the control law in CPAL, which provides native support for Finite State Machines (FSMs) to describe the logic of the algorithm, in a similar way as StateFlow. The CPAL model controls the plant model designed in the Simulink environment. At this stage, timing delays are introduced: the controller tasks are activated with input-to-output delays using timing annotations in the CPAL model (input and execution time jitter). The timing annotations are also useful for defining tasks’ periods, deadlines, priorities of execution, and the scheduling policy. The CPAL interpreter, which can be seen as an execution engine, runs the controller model within the Simulink environment that hosts the CPAL/Simulink co-simulation. The simulation results such as control performance, task activation diagram and values of the outputs are all available within Simulink.

As discussed in [10], the controller design can be done using two analytical methods: expected control performance and worst-case control performance. The Jitterbug toolbox [11] is used to calculate the expected value of quadratic control costs. The Jitter margin toolbox is used to calculate the worst-case control cost, as explained in details in Section 5.2.1. For a given control performance, this tool determines the tolerable jitter margins. In turn, these jitter margins provide admissible deadlines for the controller tasks. Using the proposed co-simulation, we verify the tolerable input jitter margin $J^{h}$ and tolerable input-to-output delay (StA delay) under which the system maintains an acceptable stability performance. We also fine-tune the obtained deadline for step response expectations when required. Further, using simulations, we study the effect of these tolerable jitter margins on control performance.

#### Step 2: Software Design

At the end of Step 1, each controller model consists of a single task performing sensing, computation and actuation. Note that this task can be integrated with other existing tasks (“Software components” block in Figure 1). At Step 2, a suitable scheduling solution, i.e., a scheduling policy and the associated parameters should be selected so as to meet the real-time constraints expressed as deadlines derived at the first step. This can be achieved, for instance, using the optimization framework in [12], a form of scheduler synthesis. Schedulability analysis has to be performed under some Worst-Case Execution Time (WCET) assumptions for all tasks. These values can be obtained by analysis or, as in our approach, approximated with on-target measurements. If this scheduling configuration meets the timing performance needed to provide the necessary control performance to the controller task then the design flow moves on to Step 3. Otherwise, we return to Step 1 and redesign the control law.

The same CPAL model executed in the simulation environment (in the previous step) is now interpreted directly on the target to measure the execution time of the task. Schedulability analysis can then be performed, and we propose a novel schedulability analysis for FIFO policy with offsets in Section 4. Although FIFO is outperformed by most policies in terms of meeting deadlines [13], it has the advantage that the scheduling order does not depend on the execution times, irrespective of the platform. The schedulability analysis checks whether the controller task we integrate with the existing software components remains schedulable or not.

This stage, if successful, ensures that the timing constraints coming from the control laws are met by the software and execution platform. If unsuccessful, we can first try to optimize the CPAL code. This may include breaking down the controller task into sub-tasks, for instance one for sensing, one for computation and one for actuation, which is a classical strategy to increase the schedulability of control systems [14], but in some cases the suitable strategy has to be specific to the application. If still unsuccessful, the process returns to Step 1 for a redesign or fine-tuning of the controller. In any cases, the model used at Step 1 for functional simulation will be the one used for execution on the target hardware.

#### Step 3: Model Introspection

From Step 2, we obtain a functional CPAL controller along with the scheduling parameters to be configured for on-target execution. These parameters have been derived from the models. To make sure that there is no distortion between the model’s assumptions and the execution, task characteristics such as period, offset, jitter, priority, deadline as well as the activation time of the current and previous instances are monitored during execution using the CPAL introspection features. In Section 5, we discuss the monitoring of CPAL model execution at run-time, especially the monitoring of timing tolerance specifications such as input jitters, output jitters and the input-to-output delays.

## 3. Analysis and Co-Simulation of Controller Design

This section explains the controller design using analytical methods and co-simulation. The result of this stage is a controller whose stability and more generally performance are guaranteed under certain assumptions on the worst-case timing behavior of the software implementation.

### 3.1. Jitter Analysis

Jitter analysis is performed using two evaluations, namely the evaluation of the expected control performance, and of the worst-case control performance. For instance, the Jitterbug toolbox [11] can be used to calculate the expected value of quadratic control costs. This measure in the general case is not sufficient to guarantee the stability of the plant [10], but stability can be verified through worst-case control performance analysis. In our framework, the technique presented in [15] and implemented in the jitter margin toolbox is used for the derivation of the jitter margins, both input and input-to-output delays, ensuring stability under the worst-case control performance. The calculated jitter margins imply the maximum deadline for a controller task. This theoretical bound on the deadline derived by analysis may be further fine-tuned by simulation as explained in the next subsections.

### 3.2. Controller Modeling in CPAL

CPAL, short for Cyber-Physical Action Language, is a modeling and discrete-event simulation language for cyber-physical systems [16]. CPAL serves as a design-exploration platform with graphical representation. The models can be executed both in simulation mode as well as in real-time mode on an embedded target. CPAL is a lightweight execution engine (around 10,000 lines of C code) designed for timing predictability that can run on top of an OS or without any OS, and thus without the interferences the OS would create.

**Figure 2:**
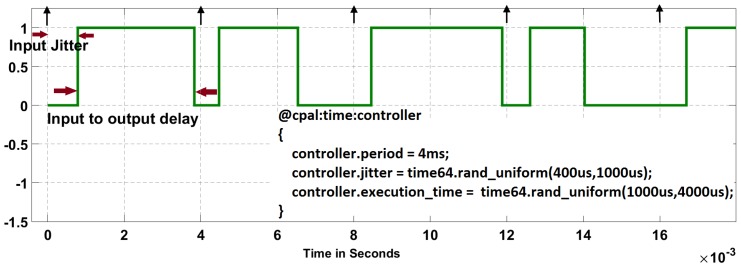
Simulating random input and output jitters affecting a CPAL controller model using timing annotations. Level 1 means that the controller is being executed.

In case of simulation, execution is as fast as possible according to a logical clock and not the physical time (see [17]). Typically, executing in simulation mode is several orders of magnitude faster than in real-time mode. The controller code executes in zero-time during simulation, except if it uses predefined CPAL timing annotations. The simulation mode CPAL interpreter is an execution engine hosted by an operating system. The simulation execution can be carried out in a stand-alone built-in simulation environment [18] or it can be used in co-simulation environments, for instance as in this work integrated in MATLAB/Simulink as an S-function. CPAL aims to achieve the same temporal behavior in simulation mode and real-time mode on the target. This property is referred to as *timing equivalence*. It can be achieved through timing annotations to inject delays in the simulation model. Figure 2 illustrates the CPAL timing annotations to inject input and output jitters in a control model.

**Figure 3:**
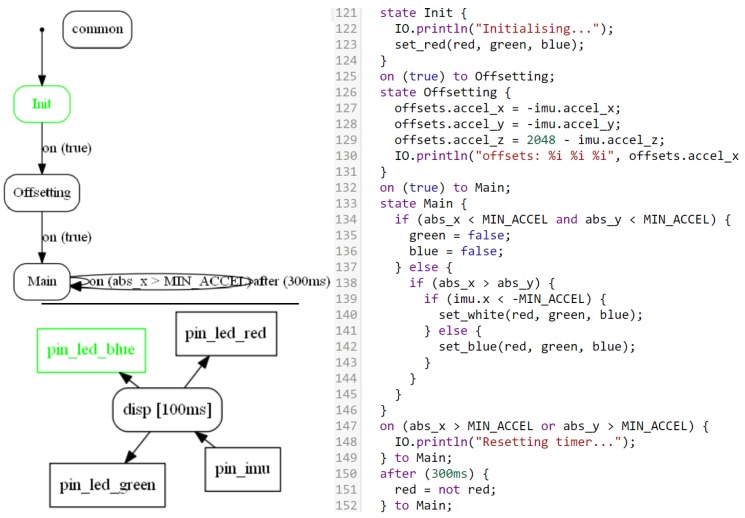
CPAL program illustrating the native support for FSM, conditional and timed state transitions. The top-left graphic is the representation of the FSM embedded in a process, while the bottom-left graphic is the functional architecture with the flows of data, as both seen in the CPAL-editor.

Like other modeling environments for control programs such as StateFlow, CPAL provides support for Finite State Machines (FSMs) with conditional and timed transitions. As can be seen in Figure 3, transitions can happen either when a boolean condition is true, after a certain time duration is spent in the active state, or the conjunction of both. A distinctive feature of CPAL is that it relies on model interpretation: a CPAL model verified by simulation can be executed directly on an embedded target such as ARM Cortex - M4 (FRDM K64F) and ARM Cortex - A7 (Raspberry Pi). Model-interpretation is well suited for rapid-prototyping [19] and prevents any distortion between models and code that could be introduced during code generation. A disadvantage of model interpretation is that it is slower than compiled code. For that reason, it is not always a practical solution for on-target execution. For the purpose of simulation on desktop machines, the execution time of the control part is however not an issue, especially in a co-simulation environment where simulating the plant is by far the most time-consuming task.

**Figure 4:**
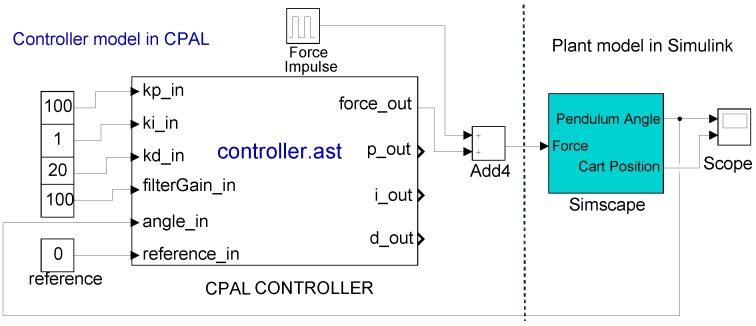
Controller model for an inverted pendulum integrated within the Simulink environment. Input data of the controller are visible in the design window and can be changed without the need to access the CPAL model. The output data are updated by the CPAL controler. The controller model is written in CPAL and executed by an interpreter embedded in the controller block. The *ast file* format is the more-compact binary equivalent form of the source-code controller model.

The CPAL documentation, a graphical editor and the execution engine for various desktop and embedded platforms are freely available at http://www.designcps.com. The CPAL control library as in Figure 4 needed to execute in MLSL controller models written in CPAL, and the models to reproduce the experiments of this paper are freely available at https://www.designcps.com/wp-content/uploads/cpal_codesign_framework.zip.

### 3.3. Co-simulation in MATLAB/Simulink

In our proposed co-simulation approach, a controller model is designed in CPAL, and the plant model in Simulink. Controllers can easily be designed in Simulink too. However, Simulink out-of-the-box is not offering possibilities to study the performance of control loops subject to scheduling and networking delays. Indeed, varying execution times, preemption delays, blocking delays, kernel overheads cannot be captured in the standard Simulink environment. This can be done only with TrueTime [2], which, to the best of our knowledge, is the most widely used tool in the real-time and control communities to study control performance subject to timing irregularities. One should also cite T-Res [4], a more recent and modular version of TrueTime.

In [5], we have discussed how to integrate the timing behaviour of the controller into Simulink models. In this work, we have studied how CPAL timing-accurate interpretation in Simulink compares against TrueTime and T-Res. The important difference, and also the advantage of our co-modelling approach is that the same model used during simulation can be used on target, whereas TrueTime and T-Res are simulation environments. Also, like Simulink, CPAL is a high-level embedded systems specific language which favors productivity and correctness by providing domain-specific constructs and abstractions [20]. In the case of the co-simulation of CPAL within MLSL, Simulink acts as the primary simulator while CPAL executes the controller model as an S-function, and is being called by the Simulink engine. The S-functions (system-functions) are high-level programming language description of a Simulink block written in C, C++ etc. The CPAL control library is implemented as a *mex* (Matlab Executable) file, which executes the CPAL controller model. This CPAL controller is a generic execution engine that can run any CPAL model. Before execution, the CPAL source model is converted into a binary-equivalent representation (an Abstract Syntax Tree, shortly ast file format) using the CPAL parser. The Simulink engine interacts with the CPAL model through data flows and control flows. Data flow, for instance *force_ out* in Figure 4, are used for the exchange of information between the Simulink engine and the CPAL controller, while the control flows define when Simulink invokes the CPAL S-function.

**Figure 5:**
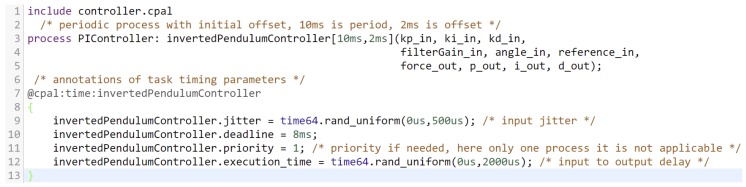
Snippet of CPAL code instantiating a controller of period 10 ms and offset 2 ms and specifying the variation of the input jitter $J^{h}$ and the input-to-output delay τ during a simulation run. This is achieved through a timing annotation executed in simulation, but ignored once on target.

The implementation is discrete-event-based simulation using Simulink built-in zero-crossing detection. The concept of tasks and real-time schedulers are available natively in CPAL. The default CPAL scheduling policy is FIFO, but CPAL also supports Non-Preemptive Earliest Deadline First (NP-EDF) and Fixed Priority Non-Preemptive (FPNP). In Figure 5, we show the instantiation of a controller task and the task parameters with the delays and jitters. A timing annotation can also specify the scheduling policy if the controller consists of several tasks. Simulation of the plant dynamics is carried-out by computing model states at successive time steps over a specified duration. This computation is done by a solver provided in Simulink. Since our overall model is discrete, a variable step size solver is used in our co-simulation approach. The rationale behind this choice is that for the timing analysis of real-time control systems, it is necessary to reduce the step size (when needed) to increase the accuracy when model states are changing rapidly during zero crossing events. Section 5.1 presents an example co-simulation of a simplified *cruise control system*.

## 4. Timing Verification Using Schedulability Analysis

The next step in the framework is the timing verification of the controller model designed in the previous step. From the jitter margins, we derive the deadlines of the controller task(s). Typically, it will be a single task, but the controller can also be implemented as several tasks such as an input task, a computation task and an output task. The deadlines will be used for the scheduler synthesis and schedulability analysis. To obtain realistic Worst-Case Execution Times (WCET) for the schedulability analysis, we use a measurement-based technique in which the controller model is executed on the target hardware.

### 4.1. Worst-Case Execution Time (WCET) Measurement

The CPAL controller model which we executed earlier in the co-simulation environment is now uploaded to the target platform to estimate the WCET by measurements. The CPAL model-interpretation engine is specific to a target platform, it can be executed on top of an Operating System (OS) or without an OS, the latter being called Bare-Metal Model Interpretation (BMMI). There are two ways to estimate the WCETs: using a logic analyzer or taking advantage of CPAL in-built execution-time measurement feature. The latter possibility is only available when CPAL is hosted by an OS, as freeRTOS, embedded Linux or Raspbian. It does not require connecting the target to an external measurement device and instrumenting the code, and thus provides a quick method to estimate the WCET. It is, however, less accurate than measurements using logic analyzer, since it involves additional run-time overhead in the interpretation engine.

For the discrete-time PID controller used in Section 5, the measured WCET of the CPAL controller task using logic analyzer is 34.4 μs on a Raspberry Pi2 model B. This can also be obtained using the in-built feature of CPAL --stats, a command-line option to be used when we execute the model on target. When we remove the code of the actual control algorithm, leaving just the skeleton of the tasks, we can observe the scheduler overhead, which amounts to 155 μs. When we execute the model as it is, we observe the scheduler overhead plus the execution time of the task to be 189 μs. The difference between these two values would then provide the execution time of the task, 34 μs, which is indeed observed also on the logic analyzer. With an ARM Cortex-A7 core at 900 MHz, Raspberry Pi is a cost-effective development platform to experiment with CPAL but it is not suited for executing real-time applications due to large timing variabilities (e.g., jitters in task release times). The best supported platform with respect to timing predictability is the NXP FRDM-K64F, a SOC on which the CPAL execution engine runs on the bare hardware, thus without any interference and latency from an OS. As provided in the Supplementary Materials (both WCET measurement and jitter measurements), we experiment the same controller model on FRDM-K64F target too, which is a BMMI target. Despite BMMI, due to inferior hardware configuration, we observe that the same task takes 340 μs to execute on the FRDM-K64F, about 10 times more than on the Raspberry Pi. We present the model on target experiments of Section 5 with Raspberry Pi because we could output the jitter measurements on the console at run-time through process introspection features. CPAL on FRDM-K64F does not have a facility to provide console outputs. In this case, a logic analyzer helps us to monitor the model executed on the target.

Deriving safe and precise WCET bounds is a difficult issue in itself (see [21] for a survey), and determining WCET estimates using state-of-the-art techniques and tools is outside of the scope of this work. Although it is a practical approach widely employed in the industry, using measurements as done in this work carries the risk of being unreliable because the worst-case situation might not have been observed. This becomes especially true for complex systems, with many tasks and architectures including multiple cores and multiple levels of caches. In such settings, more advanced WCET estimation techniques must be employed. Our framework would however work with any other WCET estimation techniques such as static deterministic analysis or probabilistic analysis. For instance, it is possible on the basis of the measurements to provision for a safety margin, typically using probabilistic arguments [22]. This margin can for instance account for cache latencies which have not been considered here. Another option is to employ an analytic WCET analysis, generally considered safer than measurement-based techniques, although much more conservative.

### 4.2. FIFO Scheduling to Simplify Design and Verification

We are interested in devising an environment that eases the design and verification of embedded real-time systems. A main goal is to provide an environment where also the inexperienced designers are able to quickly model and deploy trustworthy embedded systems without for instance having to master real-time scheduling theory and resource-sharing protocols. Especially corner case faults due to different timing behaviors or race conditions can be a nightmare to debug. We acknowledge that techniques to avoid these problems exist, but they require experience and make both the design and the code more complex and error-prone. When processing power is sufficient other concerns than performance, such as simplicity and predictability, can be considered. In our context, as shown in [13], FIFO exhibits two properties which greatly eases the verification:

- Deterministic execution order: the execution order of FIFO scheduling with offset and strictly periodic task activation is uniquely and statically determined. This means that whatever the execution platform and the task execution times, be it in simulation mode in a design environment or at run-time on the actual target, the task execution order will remain identical. Beyond the task execution order, the reading and writing events that can be observed outside the tasks occur in the same order. This property, leveraged by the CPAL design flow [16], provides a form of timing equivalent behavior between development and run-time phases which eases the implementation of the application and the verification of its timing correctness.
- Execution time sustainability: FIFO scheduling is sustainable in the tasks’ execution times, meaning that if a task set is deemed schedulable and the execution times of the tasks are reduced, the task set remains schedulable.

The latter property allows simulation as a valid technique for schedulability verification. In practice, however, the simulation time required can be unpractical if the least-common multiple of the task periods is too large. A schedulability analysis does not suffer from this limitation. In this context, we derive a schedulability analysis for FIFO scheduling on uniprocessor systems with strictly periodic task activation and tasks having release offsets. It should be noted that the use of offsets is a technique which increases the ability of FIFO to meet deadlines, no matter if the offset of a task is unique as in this work (see the experiments in [13]) or may vary, as in [23]. With offsets, FIFO becomes a candidate scheduling policy for low-memory embedded hardware with constrained run-time overheads.

We proposed in [12] a scheduling synthesis approach, where performance, hardware and functional constraints only need to be specified to derive a feasible low-level scheduling configuration. The framework proposed in this paper is compatible with any scheduling policy that guarantees that the deadlines will be met, although in the remainder of this paper, we will rely on FIFO which, as explained, facilitates the system design.

### 4.3. FIFO Schedulability Analysis

Here we present an analysis to check that the tasks will always terminate before their deadline. In the case of strictly periodic release, the release time $r_{i}^{j}$ of job $T_{i}^{j}$ is given by

(3) $$r_{i}^{j}=O_{i}+jh_{i}$$

and its absolute deadline $d_{i}^{j}$ by

(4) $$d_{i}^{j}=O_{i}+jh_{i}+D_{i}.$$

$D_{i}$ is the relative deadline, $h_{i}$ is the task’s period and $O_{i}$ is the task’s offset. Even though we are not aware of any prior work on FIFO scheduling with offsets, we were able to construct a schedulability analysis for this policy using already established schedulability results, in particular, the schedulability test for EDF with offsets presented by Pellizzoni and Lipari [24].

We note that FIFO is work-conserving in the sense that it does not introduce any idle times when work is pending. This means that prior to any deadline miss, there must be a busy period in which the processor is not idling. As we assume arbitrary offsets and strictly periodic releases, we do not know when a deadline-miss happens and so, would need to validate all busy periods within twice the hyperperiod. To avoid this prohibitively long search, we construct for each task, a hypothetical critical instant leading to a task’s first deadline miss. Let $\tau_{i}$ be the task to miss its deadline, and $\tau_{i}^{j}$ released at $r_{i}^{j}$ the corresponding job. The critical instant happens when all tasks other than $\tau_{i}$ release a job as close to $r_{i}^{j}$ as possible. If we can prove that despite this pessimistic assumption, job $\tau_{i}^{j}$ will finish before its deadline $d_{i}^{j}$, we can conclude that no job of task $\tau_{i}$ will ever miss its deadline. If we can repeat the same argumentation for each task in Γ, we can conclude that the complete task set is schedulable.

Formally, we define for each task $T_{i}$ a pseudo task-set $\hat{\Gamma }$ that represents the critical instant for task $T_{i}$. The two task sets Γ and $\hat{\Gamma }$ only differ in the task offsets, the rest of the parameters remaining identical. Let ${\hat{T}}_{i}^{j}$ be a job that misses its deadline. As we know that in a work-conserving scheduling algorithm, a deadline miss must be within a busy-period *L*, we set the release time as follows ${\hat{r}}_{i}^{j}=L$ and its deadline to ${\hat{d}}_{i}^{j}=L+D_{i}$.

George et al. [25] presented a bound based on the task deadline and the utilization of the task set:

(5) $$L_{U}:=\max_{i}\left\{D_{1},D_{2},\ldots ,D_{n},\frac{\sum_{i=1}^{n}\left(h_{i}-D_{i}\right)U_{\Gamma }}{1-U_{\Gamma }}\right\}$$

Ripoll et al. [26] presented a bound based on the following recursive equation:

(6) $$L_{R}^{a+1}:=\sum_{i=1}^{n}\frac{L_{R}^{a}}{h_{i}}C_{i}$$

Since both bounds $L_{R}$ and $L_{U}$ are independent, we can take the minimum of both as the task set’s busy period *L*:

(7) $$L:=\min\{L_{R},L_{U}\}$$

Naturally, the busy period is only bounded if the task set utilization $U_{\Gamma }$ is less than or equal to one.

We now select the task parameter of each task ${\hat{T}}_{l}$ with l≠i to maximize the likelihood of a deadline miss of job ${\hat{T}}_{i}^{j}$. To this end, we postpone the job release of the last job of task ${\hat{T}}_{l}$ executed before the deadline miss as much as possible. An earlier job release will only increase the slack time and so, reduce the pressure on the finishing time of job $T_{i}^{j}$.

In case of a higher priority task, i.e., ${\hat{T}}_{l}$ with l<i, the job must be released just before or synchronously with ${\hat{T}}_{i}^{j}$, whereas tasks with lower priority must be released strictly before ${\hat{T}}_{i}^{j}$. Since we use task priorities as a tie breaker, a lower priority task released synchronously with ${\hat{T}}_{i}$ would be executed after, and not before task ${\hat{T}}_{i}$. Pellizzoni and Lipari presented a computation of the minimum distance between any two release times of two different tasks $T_{i}$ and $T_{l}$. In contrast to their work, we are not only interested in the minimal distance, but also in the minimal distance larger than zero. We therefore repeat the computation of the minimal distance.

Let δ be distance between *j*th job of task $T_{i}$ and the *k* job of task $T_{l}$:

(8) $$\delta_{i,l}=j\cdot h_{i}+O_{i}-k\cdot h_{l}+O_{l}$$

By replacing $h_{i}$ with $x_{i}\cdot \gcd\left(h_{i},h_{l}\right)$ and $h_{l}$ with $x_{l}\cdot \gcd\left(h_{i},h_{l}\right)$, we get

$$\begin{array}{ccc} \delta_{i,l} & = & j\cdot h_{i}+O_{i}-k\cdot h_{l}+O_{l}\\ & & j\cdot x_{i}\cdot \gcd\left(h_{i},h_{l}\right)+O_{i}-k\cdot x_{l}\cdot \gcd\left(h_{i},h_{l}\right)+O_{l}\\ & & \left(j\cdot x_{i}-k\cdot x_{l}\right)\gcd\left(h_{i},h_{l}\right)+O_{i}-O_{l} \end{array}$$

Since $j\cdot x_{i}-k\cdot x_{l}$ can take any arbitrary value, we replace it by *x* and get

(9) $$\delta_{i,l}=x\cdot \gcd\left(h_{i},h_{l}\right)+O_{i}-O_{l}$$

Now, we just need to find the smallest $\delta_{i,l}\geq 0$ and the smallest $\delta_{i,l}\geq 1$, which are given by

$$x=\frac{O_{l}-O_{i}}{\gcd(h_{i},h_{l})}$$

and

$$x^{\prime }=\frac{O_{l}-O_{i}+1}{\gcd(h_{i},h_{l})}$$

Applying these values to Equation (9), we get

(10) $$\Delta_{i,l}=O_{i}-O_{l}+\left\lceil \frac{O_{l}-O_{i}}{\gcd(h_{i},h_{l})}\right\rceil \gcd\left(h_{i},h_{l}\right).$$

and

(11) $$\Delta_{i,l}^{\prime }=O_{i}-O_{l}+\left\lceil \frac{O_{l}-O_{i}+1}{\gcd(h_{i},h_{l})}\right\rceil \gcd\left(h_{i},h_{l}\right).$$

Finally, we can set the release time of the last job ${\hat{T}}_{l}^{k}$ of task ${\hat{T}}_{l}$ executed before ${\hat{T}}_{i}^{j}$ as follows:

(12) $${\hat{r}}_{l}^{k}=\left\{\begin{array}{cc} {\hat{r}}_{i}^{j}-\Delta_{i,l} & \;\mathrm{if}\;l\leq i\\ {\hat{r}}_{i}^{j}-\Delta_{i,l}^{\prime } & \;\mathrm{if}\;l>i. \end{array}\right.$$

The offset of task $\tau_{i}$ is given by

(13) $${\hat{O}}_{i}={\hat{r}}_{i}^{j}\;\mathrm{mod}\;h_{i},$$

and for all other tasks l≠i by

(14) $${\hat{O}}_{l}={\hat{r}}_{l}^{k}\;\mathrm{mod}\;h_{l}.$$

The remaining task set parameters, i.e., the relative deadline, period and execution time remain unchanged.

It is sufficient to validate the schedulability of $\hat{\Gamma }$: if ${\hat{T}}_{i}$ in $\hat{\Gamma }$ is schedulable with FIFO, so is $T_{i}$ in Γ. Furthermore, since we know which job of task ${\hat{T}}_{i}$ will miss its deadline in case of a deadline miss, it is sufficient to concentrate on the *j*th job ${\hat{T}}_{i}^{j}$, which allows us to reduce the analysis time. If we are able to prove or disprove a deadline miss of job ${\hat{T}}_{i}^{j}$, we can immediately abort the schedulability analysis of task $T_{i}$. Consequently, we concentrate only on job ${\hat{T}}_{i}^{j}$ and ignore all others. First, we define the number of job releases that may postpone the completion of task *i* within a given time interval.

The function $\eta_{l}^{inc}\left(t_{1},t_{2}\right)$ denotes the number of job releases of task $\tau_{l}$ within the time interval $\left[t_{1}:t_{2}\right]$, i.e., including $t_{2}$ and is given as follows:

(15) $$\eta_{l}^{inc}\left(t_{1},t_{2}\right)=\left\lfloor \frac{t_{2}-{\hat{O}}_{l}}{h_{l}}\right\rfloor +1-\left\lceil \frac{t_{1}-{\hat{O}}_{l}}{h_{l}}\right\rceil$$

The function $\eta_{l}^{exc}\left(t_{1},t_{2}\right)$ denotes the number of job arrivals of task $\tau_{j}$ within the time interval $\left[t_{1}:t_{2}\right)$, i.e., excluding $t_{2}$ and is given as follows:

(16) $$\eta_{l}^{exc}\left(t_{1},t_{2}\right)=\left\lceil \frac{t_{2}-{\hat{O}}_{l}}{h_{l}}\right\rceil -\left\lceil \frac{t_{1}-{\hat{O}}_{l}}{h_{l}}\right\rceil$$

Using these two functions, we define the processor demand PD within time interval $\left[t_{1}:t_{2}\right]$ that can delay the completion of a job of task ${\hat{T}}_{i}$ released at $t_{2}$:

(17) $$PD\left(t_{1},t_{2},i\right)=\sum_{l\leq i}\eta_{l}^{inc}\left(t_{1},t_{2}\right)\cdot C_{l}+\sum_{l>i}\eta_{l}^{exc}\left(t_{1},t_{2}\right)\cdot C_{l}$$

Again, we distinguish between tasks with higher priorities and tasks with lower priorities to correctly account for the tie-breaking policy in case of synchronous job arrivals.

We can test for a deadline miss of job ${\hat{T}}_{i}^{j}$ as follows:

(18) $$\forall t\in \left[0:{\hat{r}}_{i}^{j},i\right]:PD\left(t,{\hat{r}}_{i}^{j},i\right)\leq {\hat{d}}_{i}^{j}-t\Rightarrow {\hat{f}}_{i}^{j}\leq {\hat{d}}_{i}^{j}$$

To reduce the number of test, we observe that $PD(t_{1},t_{2},i)$ only changes at the time of a job release, which means that we only need to validate the schedulability at these points:

(19) $$Q=\{t|\exists l,k:t=k\cdot h_{l}+{\hat{O}}_{l}\wedge t\leq L-D_{i}\}$$

Hence, we can validate the schedulability of task $T_{i}$ as follows:

(20) $$\forall t\in Q:PD\left(t,{\hat{r}}_{i}^{j},i\right)\leq {\hat{d}}_{i}^{j}-t\Rightarrow {\hat{f}}_{i}^{j}\leq {\hat{d}}_{i}^{j}$$

We note that the schedulability test is sufficient but not necessary, and does not provide an equivalence between the schedulability of Γ and $\hat{\Gamma }$. The schedulability analysis can falsely deem a schedulable task set unschedulable, but not the inverse.

From Equation (20), we find the worst-case finishing time of the task $T_{i}$

(21) $${\hat{f}}_{i}=\max_{\forall t\in Q}\left\{PD\left(t,{\hat{r}}_{i}^{j},new\right)+t\right\}$$

Then the worst-case response time of a task $T_{i}$ is $R_{i}^{w}$

(22) $$R_{i}^{w}={\hat{f}}_{i}-{\hat{r}}_{i}$$

**Algorithm 1** Worst-Case Response time $R_{i}^{w}$1:i=12:isSchedulable = true3:L= computeBusyPeriod4:**while** i≤n∧ isSchedulable **do**5:    ${\hat{r}}_{i}^{j}=L$6:    ${\hat{O}}_{i}=r_{i}^{j}\;\mathrm{mod}\;h_{i}$7:    **for all l do**8:        ${\hat{dist}}_{i,l}=$ computeMinDistance (i,l)9:        ${\hat{O}}_{l}=r_{i}^{j}-{\hat{dist}}_{i,l}\;\mathrm{mod}\;h_{i}$10:    **end for**11:    $Q=\{t|\exists l,k:t=k\cdot h_{l}+{\hat{O}}_{l}\wedge t\leq L\}$12:    **for all** t∈Q **do**13:        **if** $PD\left(t,{\hat{r}}_{i}^{j},i\right)-t>{\hat{d}}_{i}^{j}$ **then** isSchedulable = false14:        **end if** 15:        **if** isSchedulable **then break**16:        **end if** 17:        ${\hat{f}}_{i}^{j}=\left\{PD\left(t,{\hat{r}}_{i}^{j},i\right)+t\right\}$18:    **end for**19:    ${\hat{f}}_{i}=\max\left\{{\hat{f}}_{i}^{j}\right\}$20:    $R_{i}^{w}={\hat{f}}_{i}-{\hat{r}}_{i}$21:    i=i+122:**end while**23:**return** isSchedulable24:**return** $R_{i}^{w}$

Algorithm 1 consolidates the analysis presented so far. Using this algorithm, we can derive the worst-case response times of all task. To check schedulability, we verify that these response times are less than or equal to the fine-tuned deadlines, which we have obtained from the previous step. To achieve transparency and to ease the reproduction of the results, the source code of the programs used in our experiments, including the schedulability test, is available online (https://www.designcps.com/wp-content/uploads/cpal_codesign_framework.zip). The source code enables the reproduction of the experiments presented in this paper, as well as evaluation for different parameters settings. The tool *cpal2x* (see [17] for usage), which is available in the CPAL distribution, extracts the timing information (timing and scheduling annotations) from the controller function designed at Step 1. This constitutes the system task model which is then inputted to the presented schedulability analysis.

## 5. Evaluation and Results

We now evaluate the framework with the help of an automotive control system. Before presenting the evaluation, we describe the system model. As depicted in the framework of Section 2, the evaluation consists of three steps. Firstly, we calculate the tolerable jitter margin values under which the system remains stable using jitter margin analysis. The calculated output jitter margin provides the maximum deadline for the controller task. This deadline is further fine-tuned using co-simulation that provides additional and more fine-grained information about the control performance. Secondly, we evaluate the schedulability of the controller task when executed with other tasks in the system. Finally, in the third step, we use CPAL introspection to check that the run-time behavior of the controller task complies with the design assumptions.

### 5.1. Motivating Example : Cruise Control ECU

**Figure 6:**
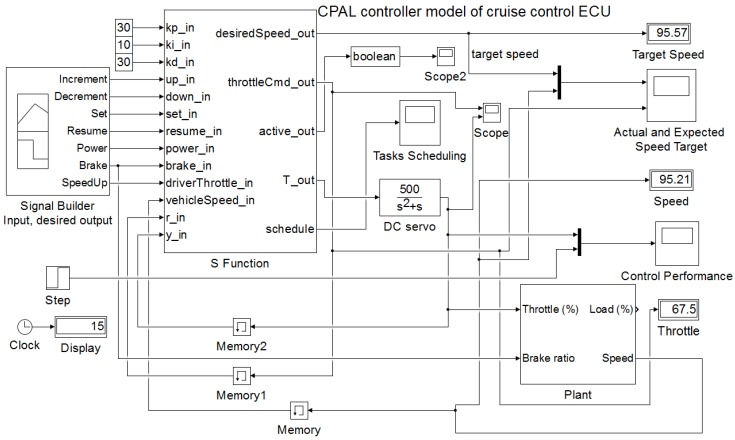
Illustration of a CPAL controller in Simulink. Here, the CPAL model controls the servo which in turn actuates the engine throttle. The controller task is executed with simulated input-to-output delays.

A Cruise Control system maintains the speed of a car at a desired level. For that, the system uses a servo mechanism that takes over the throttle of the car to maintain a steady speed as set by the driver. The system model used is taken from the Simulink reference examples [27], but the controller model is replaced by a CPAL implementation. Without injecting run-time delays, both the CPAL implemented version of the controller and the Simulink version provide the same outputs. This comparison is available in the Supplementary Materials provided. Figure 6 shows the architecture of the co-simulation model. The proposed co-simulation environment provides the control performance with the run-time delays due to execution times and interferences from higher priority tasks, and facilities the visualization of the task scheduling. In addition, the same controller model developed for simulation can be executed on the target by the CPAL execution engine.

In our implementation, different tasks and variables are defined within the controller model. We consider tasks, namely *set point manager*, *cruise control manager* and *sensors manager*. The label T_out in Figure 6 is the controller tasks’ output which actuates the DC servo mechanism controlling the throttle valve. We model the DC servo with the transfer function $P\left(s\right)=\frac{500}{\left(s^{2}+s\right)}$. The controller developed relies on a PID control algorithm with proportional gain $K_{p}=0.96$, derivative gain $K_{d}=0.049$, integral gain $K_{i}=0.12$ and filter divisor N=5.0.

### 5.2. Controller Design

The evaluation of the controller designed consists of two steps, namely the analytical jitter margin method and the co-simulation technique.

#### 5.2.1. (Step 1. a) Stability Verification Using Jitter Margin Concept

**Figure 7:**
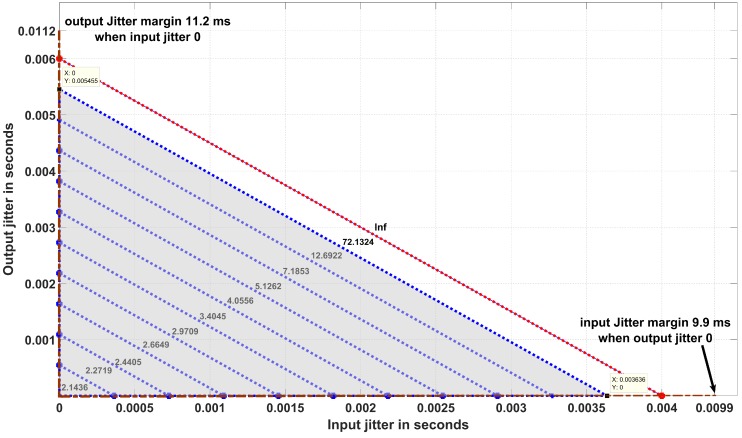
The worst-case control cost calculation during various input and output jitter occurrences. The control cost mentioned here is $H_{\infty }$, a gain parameter. Finite control costs indicate that the system is stable while an infinite value ${}^{\prime }Inf^{\prime }$ indicates that the system tends to be unstable. At zero input and zero output jitter, the highest performance is achieved. The control cost increases when jitters increase.

For a given controller and a nominal input-to-output delay L, the Jitter margin toolbox [15] computes the tolerable level of jitter for which stability is guaranteed (like phase margin and gain margin computations of control systems). This toolbox provides the stability curve that determines the maximum tolerable output jitter $J^{\tau }$ and maximum tolerable input jitter $J^{h}$, based on the nominal input-output delay L. Figure 7 shows the worst case control cost which is a $H_{\infty }$ (H-infinity) performance metric calculated for different input and output jitters. For example, for the PID controller of the previous sub-section with a sampling period of 12 ms, the nominal (minimum) input-to-output delay L is equal to 5.6 ms, the input jitter margin $J^{h}$ is 3.64 ms, the output jitter margin $J^{\tau }$ is 5.45 ms, while the control cost $H_{\infty }$ is 72.13 ms. The input-to-output delay, which is the sum of encountered jitters during the execution of the controller task is then 9.09 ms. The control cost we use is $H_{\infty }$, a gain parameter calculated when we apply a disturbance input to the plant and the corresponding output amplifies.

When we get a finite value for the gain parameter $H_{\infty }$, it indicates that the system remains stable. Beyond the jitter margin, we observe that the gain becomes infinite, which means that the system tends to be unstable. During the ideal situation where the controller task executes with zero input jitter and zero output jitter, we obtain the highest possible control performance with control costs $H_{\infty }$ equal to 2.14. If we want to guarantee a certain control performance, expressed in terms of $H_{\infty }$, we have to design the system such that the experienced jitters are within the jitter margins leading to $H_{\infty }$ being no greater than the target. For instance in Figure 7, the jitters must remain in the shaded region to ensure that $H_{\infty }$ remains equal to 72.13. This allows to deduce that the controller task deadline must be less than 9.09 ms. Now, to fine-tune the deadline and also to study the effect of scheduling choices on the control performance, the co-simulation approach is used. In the implementation of the cruise-control system, the controller task, denoted Task 1, is activated every 12 ms. Task 2 is another task with the same period, always activated before Task 1. In case both are released at the same time, the execution time generates an input jitter for Task 1. This latency, plus the varying execution time of Task 1 itself, induce the output jitter.

**Figure 8:**
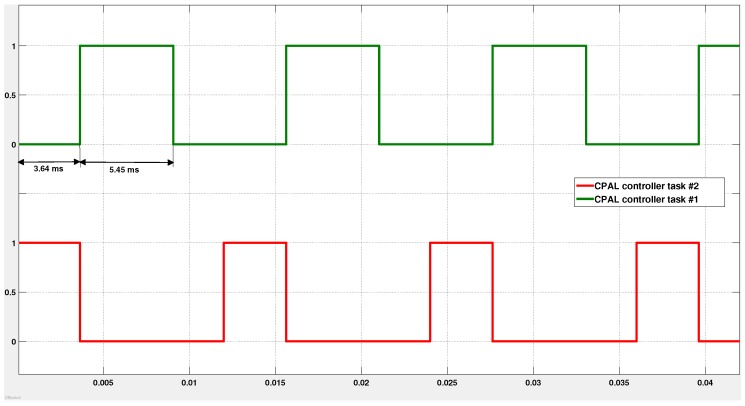
Successive activations of two tasks under FIFO. Task 1 is the controller we design with a period of 12 ms. Task 2 is the *cruise control manager* also with a 12 ms period. As Task 2 is of higher priority, it is activated first when both tasks are released simultaneously. Using varying execution time annotations for Task 1 and Task 2, we enforce an input-to-output delay of at most 9.09 ms for Task 1, which is the bound obtained from jitter margin analysis.

**Figure 9:**
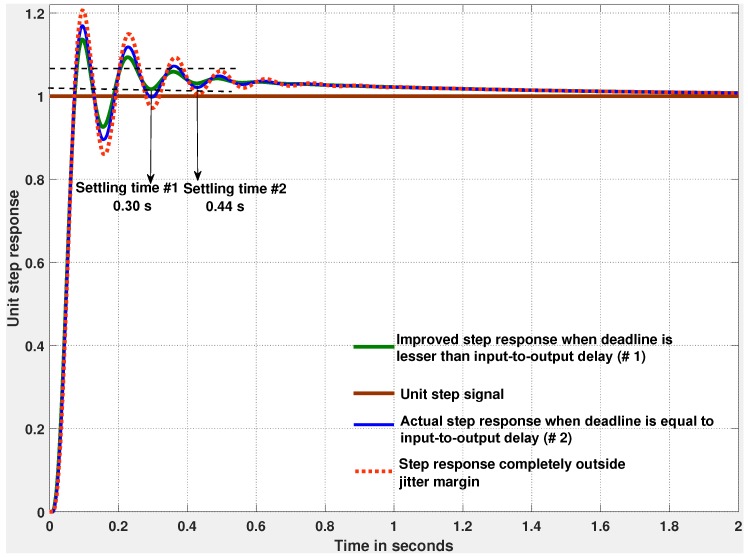
Control performance using step response for different deadline assignments: equal, less and greater than the input-to-output delay (resp. blue, green and red curves). The green curve (reduced overshoot one) is obtained with a deadline value equal to 8.2 ms chosen such that the settling time within 2% of the steady-state value is less than 0.3 s. When the control task deadline is greater than the jitter margin, logically the system performs poorer with increased oscillations and overshoots.

Figure 8 illustrates the execution of the tasks under First-In First-Out (FIFO) policy. As we can see from Figure 8, Task 1, the controller under design, experiences an input jitter of 3.64 ms. This is realized by means of an execution time annotation (see Section 3.3) of an interfering task activated immediately before. By setting the execution time of Task 1 to 5.45 ms, combined with the input jitter, we enforce an input-to-output delay of 9.09 ms. This is the tolerance level beyond which the system performance degrades significantly, as shown in Figure 9.

#### 5.2.2. (Step 1. b) Co-Simulation CPAL/Simulink

The co-simulation of CPAL in the Simulink environment serves two purposes: fine-tuning of the deadline and selection of the scheduling policy. Although the jitter bound derived by jitter margin analysis helps to assign the deadline, in practice a system designer may want to evaluate the control performance with the response to an input elementary signal such as impulse or a step signal. For this purpose, we feed an unit step signal in the co-simulation model to study the step response of the system. Based on the step response characteristics such as rise time, settling time and overshoot, we can decide whether a fine tuning of the deadline is necessary. For instance, if the control requirement is to achieve a desired settling time, defined as the time taken to settle within 2% of the steady state value, equal to 0.3 s, then the deadline should be no greater than 8.2 ms (versus 0.44 s with a deadline of 9.09 ms). In our previous work [5], we have exemplified the co-simulation of CPAL in Simulink to study the control system performance for different scheduling options.

### 5.3. (Step 2) Software Design

As explained earlier in Section 4.1, the controller model we designed at step 1 is now uploaded on the target platform to estimate a WCET bound. For the specifications of the controller with the sampling period of 12 ms (see Section 5.1), the execution time of the CPAL controller task measured using a logic analyzer is 34.4 μs. As explained in Section 4, WCET estimation can also be conveniently performed using the CPAL in-built --stats feature. For the controller task developed, the maximum execution time value observed is around 200 μs including the scheduler overhead. When there are no preemptions as here, or a bounded number of preemptions, it is possible to include the scheduler overhead in the WCET of the task. To provision for a safety margin, we consider the execution time along with the scheduler overhead. We use this WCET and the admissible deadline of 8.2 ms obtained from the previous step to test the schedulability of the system. The schedulability analysis presented in Section 4.3 tells us whether the integrated task set (the controller under design plus the existing tasks on the ECU) is feasible or not. In our experimental setup, the task set passes the schedulability test.

**Figure 10:**
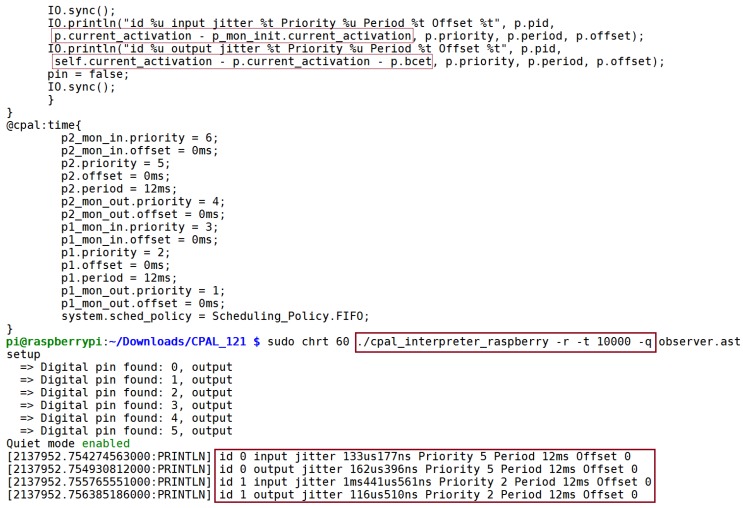
Code snippet of the two monitoring processes, including their scheduling parameters.

Once we obtain a stable controller model, we verify its run-time behavior on the target hardware. At run-time, it is possible in CPAL for a process instance to query its id, period, offset, both input and output jitters, priority, deadline and the activation times of the current and previous activations. Statistics can be collected and analyzed off-line, but it is also possible to visualize at run-time the variation of these quantities. Figure 10 shows a snippet of the code of the two monitoring tasks of Task 1, one for the input jitter and one for the output jitter, as well as their scheduling parameters. Here the choice has been made to have external tasks monitoring the jitters in order to not clutter the controller code. Although FIFO is the scheduling policy, simultaneous task releases are broken with the *priority* attribute (see Figure 10).

### 5.4. (Step 3) Introspection Features for Run-Time Verification

During model on target execution, the run-time monitoring tasks are respectively executed before the start of the controller task and immediately after. This can be for instance ensured by setting the priority attribute so that the input-monitoring process is at a higher priority than the controller task (3 in our case), while the output-monitoring task is at the immediate lower priority (1 in our case). The controller Task 2 is the *cruise control manager* of higher functional importance. It is activated first when both Task 1 and Task 2 are released simultaneously. The lower part of Figure 10 shows a sample console display of the input and output jitters during command-line execution in real-time mode (i.e., option −r in the command line) of the CPAL controller model with quiet option −q enabled. Here, jitters are recorded for 10 s on a Raspberry Pi2 model B with an ARM Cortex A7 processor.

**Figure 11:**
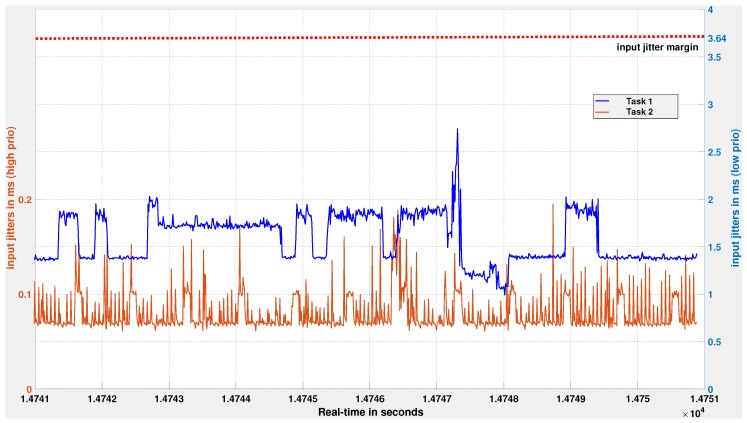
Global view of input jitter measurements of Task 1 and Task 2. The input jitter $J^{h}$ of Task 1 (blue curve, right y-axis) varies over time below 2 ms, except in rare cases where it reaches 2.7 ms. The input jitter of Task 2 (red curve, left y-axis) is bounded by 0.2 ms. The design assumption of input jitters for Task 1 is less than 3.64 ms is met by the implementation.

**Figure 12:**
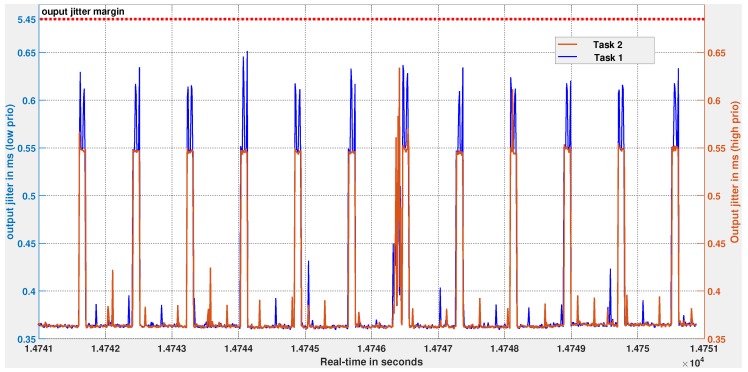
Global view of output jitter measurements of Task 1 and Task 2. The output jitter $J^{\tau }$ of Task 1 (blue curve, left y-axis) varies over time but remains below 0.65 ms. The output jitter of Task 2 (red curve, right y-axis) is bounded by 0.63 ms. The design assumption of output jitters for Task 1 is less than 5.45 ms is met by the implementation.

Figure 11 shows the input jitter measurements of the two controller tasks, Task 1 and Task 2, over a duration of 10 s. Even if Task 1 suffers delays from Task 2, we observe that its input jitters are well within the input jitter margin value of 3.64 ms (see Section 5.2.1). Likewise, Figure 12 shows the output jitter measurements for both controllers, and the cruise-control system meets the 5.45 ms output-jitter margin. These experiments, along with logic analyzer measurements confirm the design assumptions related to jitters. The logic analyzer set-up and captures files are available as additional references within the Supplementary Materials provided with this article.

**Figure 13:**
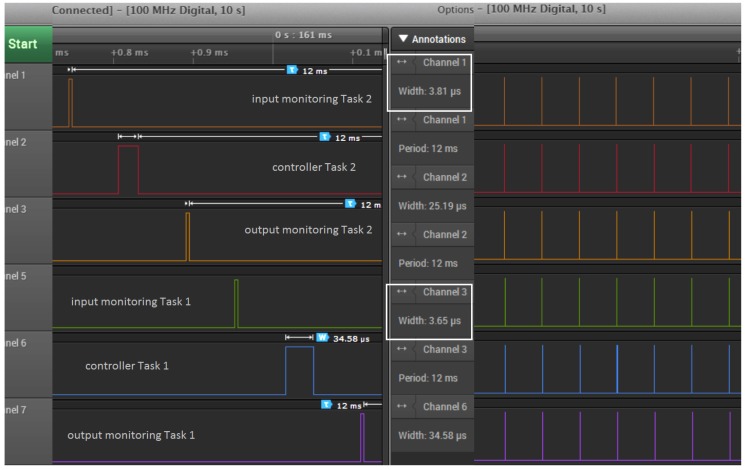
The input and output monitoring task activations for two controller tasks captured using a logic analyzer. Both the controller tasks *Task* 1, *Task* 2 are activated with a period of 12 ms. Both are different control algorithms which run for an execution time of 34.58 μs and 25.19 μs, respectively at the highlighted job instant. The monitoring tasks execute only a fraction of the controller’s computation time, typically less than 4 μs.

We enabled −q (quiet) option during model execution to get only the necessary console outputs, which are the jitter values during run-time. We record these jitter values for the purpose of visualization and to study whether the jitters are within the margins. To cross-check, we also measure the jitters using a logic analyzer with a 100 MHz sampling rate for about 10 s, as shown in Figure 13. For a particular job instance (zoomed portion of the figure), we measure an execution time of 34.58 μs for controller Task 1 and 25.19 μs for controller Task 2, which both run with a period of 12 ms. The monitoring processes (input and output) are here to help measure the input jitters, output jitters and input-to-output delay. We observe that when we do not include the printing of jitter values on the console, both input monitor and output monitor tasks (i.e., channels 1, 3 for Task 2 and channels 5, 7 for Task 1) consume less than 4 μs. Note that these monitoring tasks can be removed for the production code once the design is finalized to avoid overhead.

## 6. Related Works

In the literature of computing and control, there have been numerous studies on the effects of timing irregularities on control performance [2,3,4,5]. Cervin et al. coined the term *jitter margin* in [28], where the authors considered the output jitter margin under which the system still maintains its stability. In a subsequent work [15], Cervin extended the analysis to account for both the input and output jitters on the control performance of linear sampled-data control systems. In this paper, we integrate this analysis in a tool-supported design flow which guarantees the control performance on a given execution platform.

A technical contribution needed in this work is a FIFO schedulability analysis for periodic tasks with offsets. Closely related are the results by George and Minet published in [29], who proposed a scheduling analysis for FIFO on a distributed system assuming sporadic task releases, and the results by Leontyev and Anderson [30], who developed a tardiness analysis for FIFO scheduling of soft real-time tasks, also assuming a distributed system and sporadic task releases. The two latter works did not apply directly to our task model, i.e., periodic task with release offsets.

Sangiovanni-Vincentelli et al. discussed various methodologies to address the system design challenges in [31]. This work highlights the importance of *Assume/Guarantee* contracts during component design and explains how a contract can be applied to the design of a water flow control system. Derler et al. proposed in [7] that implicit timing assumptions are made explicit using design contracts to facilitate the interaction and communication between control and software domains. The authors discussed the support for timing-contracts-based designs using Ptolemy and Simulink. Benveniste et al. proposed in [32] to apply contracts to design methodologies. Importantly, the authors explained the mathematical concepts and operations necessary for the contract framework. All these works mentioned in this paragraph focused on the fundamental framework for design contracts, such as contract algebra applied in system design, and timing contract visualization in modeling environment. In this work, we are concerned with the application of timing tolerance contract in our Model-Based Design flow used to develop control software, thus focusing on scheduling and implementation issues.

In terms of related design environments, we identify two approaches with associated tools aiming to support control system design considering the influence of scheduling strategies:

- *TrueTime*: this MATLAB/Simulink-based tool [2] enables the simulation of the temporal behavior of controller tasks executed on a multitasking real-time kernel. In TrueTime, it is possible to evaluate the performance of control loops subject to the latencies of the implementation. TrueTime offers a configurable kernel block, network blocks, protocol-independent send and receive blocks and a battery block. These blocks are Simulink S-functions written in C++. TrueTime is an event-based simulation using zero-crossing functions. The tasks are used to model the execution of user code and are written as code segments in a MATLAB script or in C++. It models a number of code statements that are executed sequentially.
- *T-Res*: this more recent tool [4] is also developed using a set of custom Simulink blocks created to simulate timing delays dependent on code execution, scheduling of tasks and communication latencies, and verifying their impact on the performance of control software. T-Res is inspired from TrueTime and provides a more modular approach to the design of controller models enabling to define the controller code independently from the model of the task.

These tools and methods focus on simulation and analysis. They both help the designer to study the control system performance under the effects of timing delays. The system designer then takes simulation analysis results into account to develop the embedded control algorithms in the next steps. This increases the possibility of distortions between the simulation model and the implementation. An advantage of our co-simulation modelling approach is that the same controller model used to evaluate the control performance during design phase can be re-used directly on the target hardware (in the coding and testing phase) to implement the system. As discussed in our previous work [5,19], the reduced development cycle favors efficient interactions between control and software engineers. The reader is referred to [5] for a review of CPAL in Simulink, TrueTime and T-Res development environments.

## 7. Conclusion and Future Work

The timing behavior of control tasks is a critical concern in real-time digital controllers. The delays, such as input jitters, or missed executions due to temporary overload, affect system performance and are to be accounted for in the design phase. Model-driven engineering has been successful for capturing the functional requirements during design, but non-functional requirements such as timing have been traditionally overlooked. This leads to a late verification of controller timing and, in the best case, to corrections at a stage when they are costlier. This work is a contribution towards conceiving a design environment for embedded control systems that capture all the necessary functional and non-functional requirements, while providing analysis, simulation and run-time capabilities.

In this paper, we presented a framework based on timing tolerance contracts which fuses the stability and scheduling viewpoints during controller design. The three steps of the framework have been described: controller design verified by stability analysis and co-simulation, software design verified by schedulability and WCET estimation, and lastly, the implementation checked through run-time verification. The crucial advantage of our co-simulation approach based on model interpretation is that the same controller model verified in the design phase can be ported directly (without the need for code generation) to target hardware to implement the final system. This feature will ease the deployment and the update of code on distributed nodes, for instance in Industrial Internet of things (IIoT) applications.

To exhibit the framework flow, we have presented the scheduling viewpoint using novel FIFO schedulability analysis for periodic task activations with offsets. As future work, we plan to extend the framework to other schedulability analyses using tools such as Cheddar [33] and MAST [34] to support more scheduling options during scheduler synthesis. Another objective is to extend the approach to other important non-functional properties, foremost power consumption for next-generation Cyber-Physical Systems, which will require both analysis and modeling language support.

## Figures and Tables

**Table 1:** Notations used in the paper.

task-set	$\Gamma =\{T_{1},\ldots ,T_{n}\}$
pseudo task-set	$\Gamma =\{{\hat{T}}_{1},\ldots ,{\hat{T}}_{n}\}$
number of tasks	*n* ϵ *N*
job index	i,j ϵ *N*
task worst-case execution time with no interference	$C_{i}$ ϵ *R*
task period	$h_{i}$ ϵ *R*
task relative deadline	$D_{i}$ ϵ *R*
task absolute deadline	$d_{i}$ ϵ *R*
task release time	$r_{i}$ ϵ *R*
task finish time	$f_{i}$ ϵ *R*
task worst-case response time	$R_{i}^{w}$ ϵ *R*
task best-case response time	$R_{i}^{b}$ ϵ *R*
task processor demand	$PD_{i}$ ϵ *R*
task busy-period	*L*ϵ *R*
input jitter also known as sampling jitter	$J^{h}$ ϵ *R*
output jitter also known as response-time jitter	$J^{\tau }$ ϵ *R*
input-to-output delay also known as StA latency	τ ϵ *R*
k-th sensing time instance	$t_{k}^{s}$ ϵ *R*
k-th actuation time instance	$t_{k}^{a}$ ϵ *R*
nominal input-output delay	L ϵ *R*

## Supplementary Materials

The following are available online at http://www.mdpi.com/1424-8220/18/2/628/s1.

## Abbreviations

The following abbreviations are used in this manuscript:

BET	Bounded Execution Time contract
CPAL	Cyber-Physical Action Language
CPS	Cyber-Physical Systems
ECU	Electronic Control Unit
FIFO	First In First Out
LET	Logical Execution Time contract
MDE	Model-Driven Engineering
PID	Proportional Integral Differential
SDLC	Software Development Life Cycle
StA	Sensing to Actuation Delay
SWC	Software Component
TOL	Timing Tolerance contract
WCET	Worst-Case Execution Time
ZET	Zero Execution Time contract
