# Supplementary figures and images for: A Model-Driven Co-Design Framework for Fusing Control and Scheduling Viewpoints
Source: Sensors (Basel). 2018 Feb 20;18(2):628. doi: 10.3390/s18020628 (PMC5856116; doi:10.3390/s18020628)

# CPAL controller model of cruise control ECU

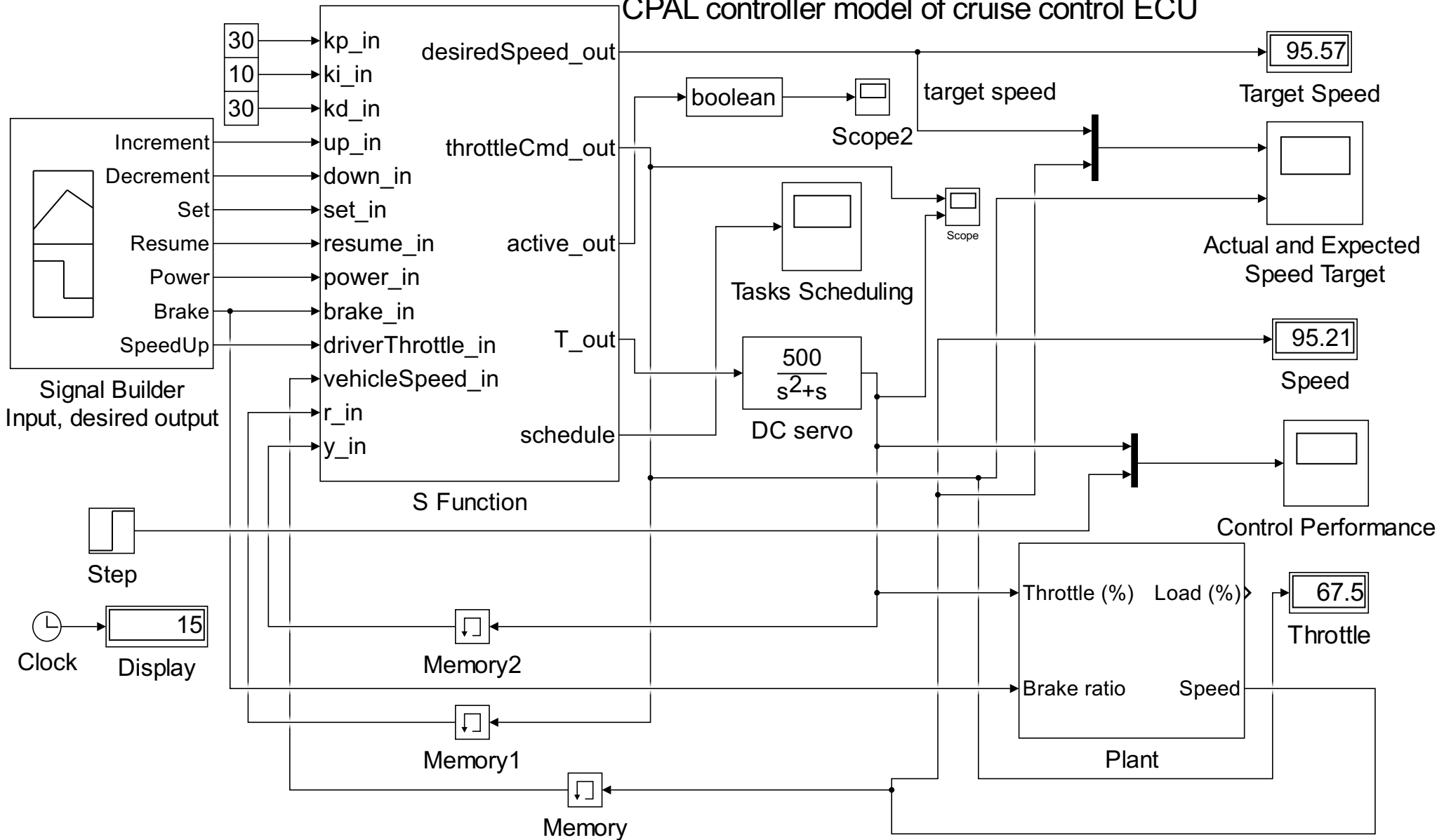

Supplement: Supplementary file 1 [file sensors-18-00628-s001.zip › cpal_codesign_framework/3_cruise/model.pdf]

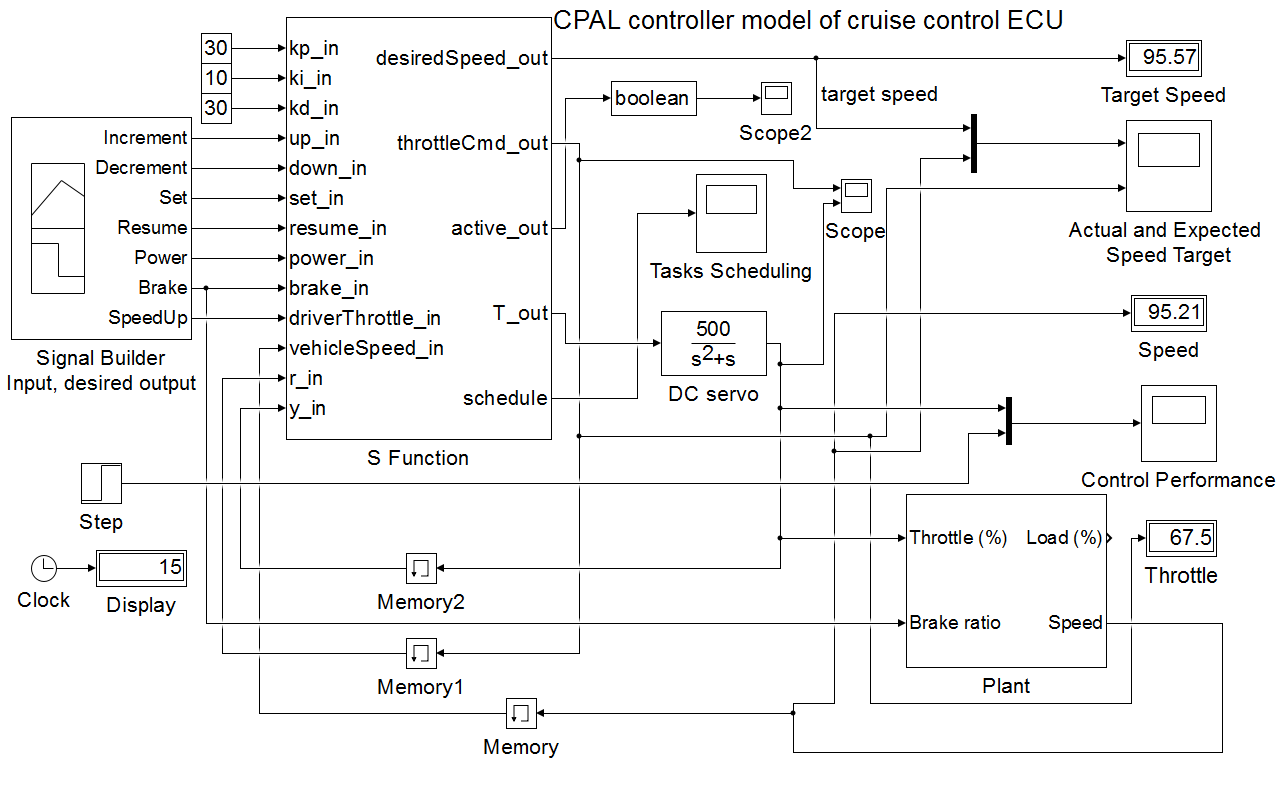

Supplement: Supplementary file 1 [file sensors-18-00628-s001.zip › cpal_codesign_framework/3_cruise/model.png]

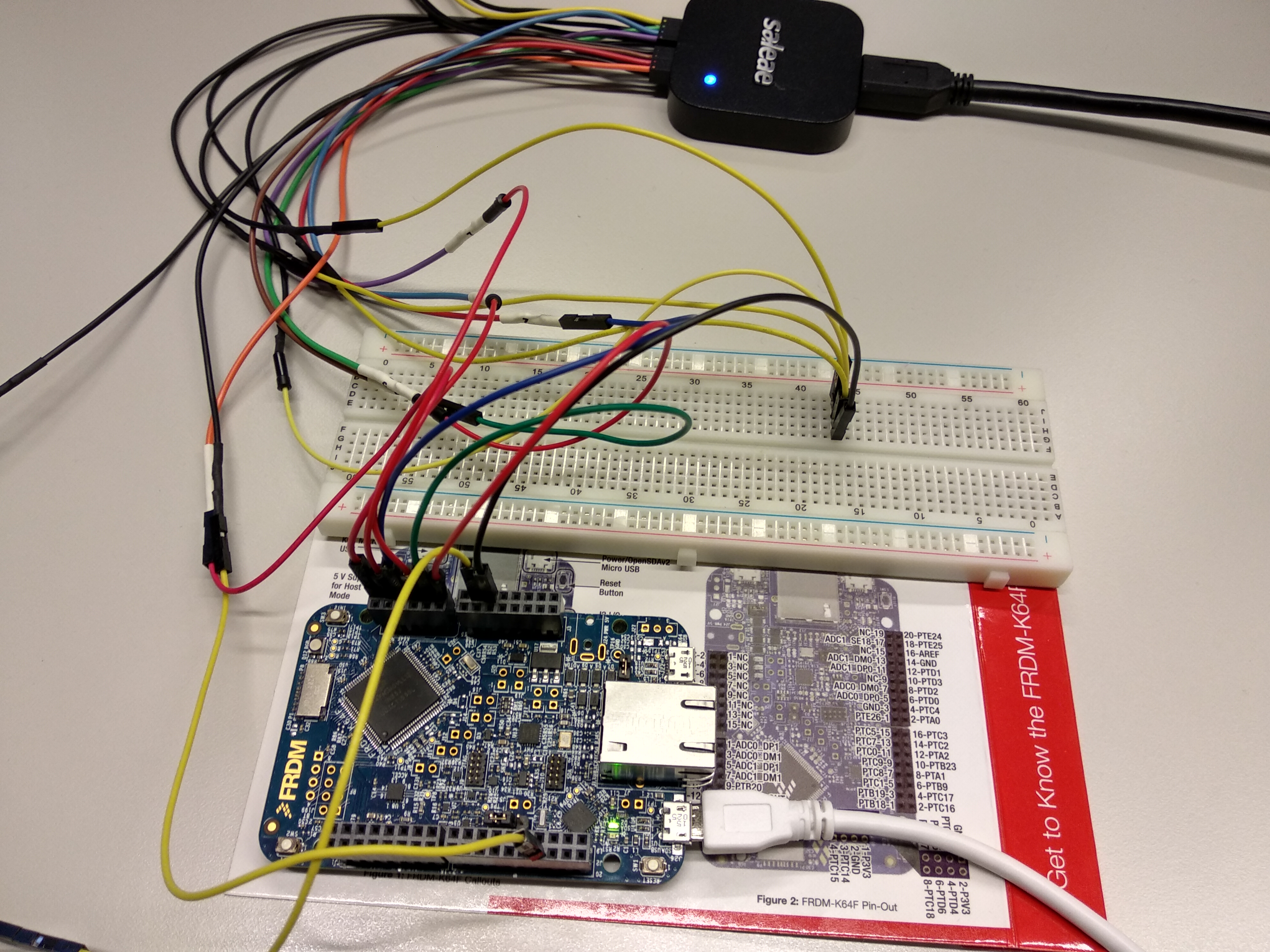

Supplement: Supplementary file 1 [file sensors-18-00628-s001.zip › cpal_codesign_framework/4_introspection_bare_metal_FRDM/BMMI_CPAL_FRDM.jpg]

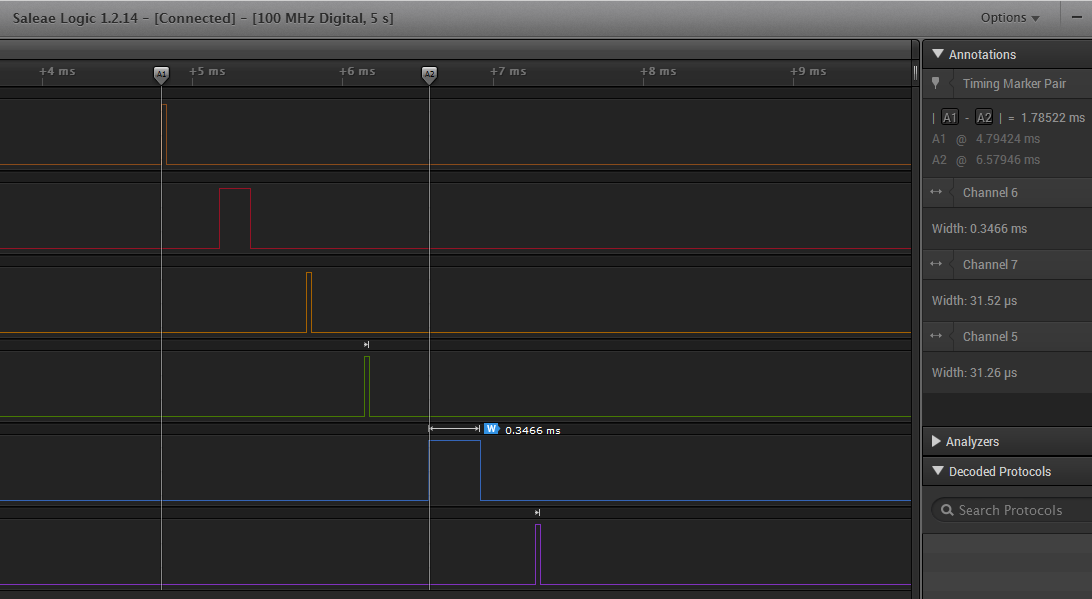

Supplement: Supplementary file 1 [file sensors-18-00628-s001.zip › cpal_codesign_framework/4_introspection_bare_metal_FRDM/frdm_all_tasks.png]

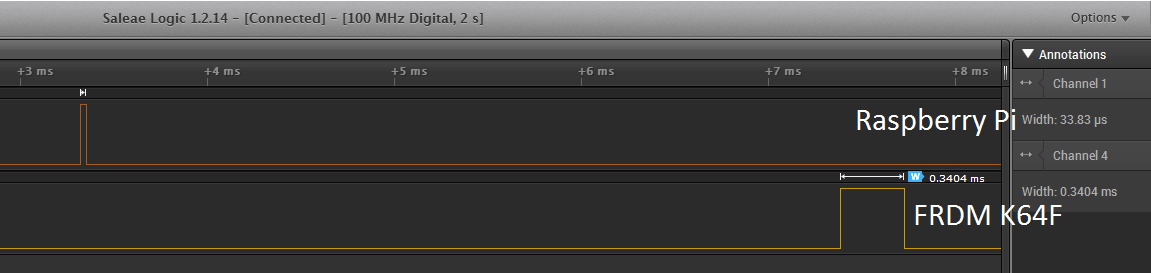

Supplement: Supplementary file 1 [file sensors-18-00628-s001.zip › cpal_codesign_framework/4_introspection_bare_metal_FRDM/WCET.png]

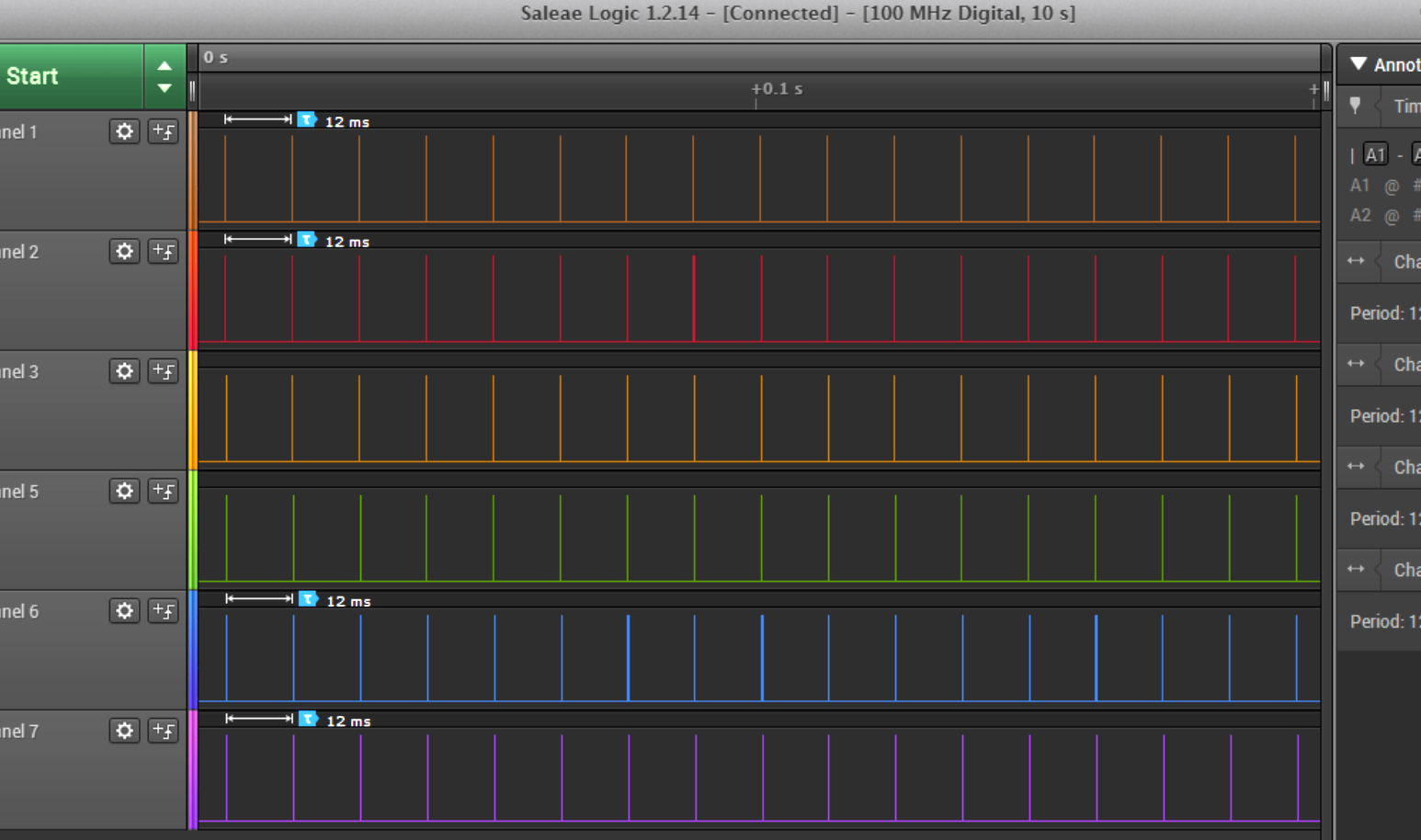

Supplement: Supplementary file 1 [file sensors-18-00628-s001.zip › cpal_codesign_framework/5_introspection_on_target_Raspberry_Pi/01_over_all_1.png]

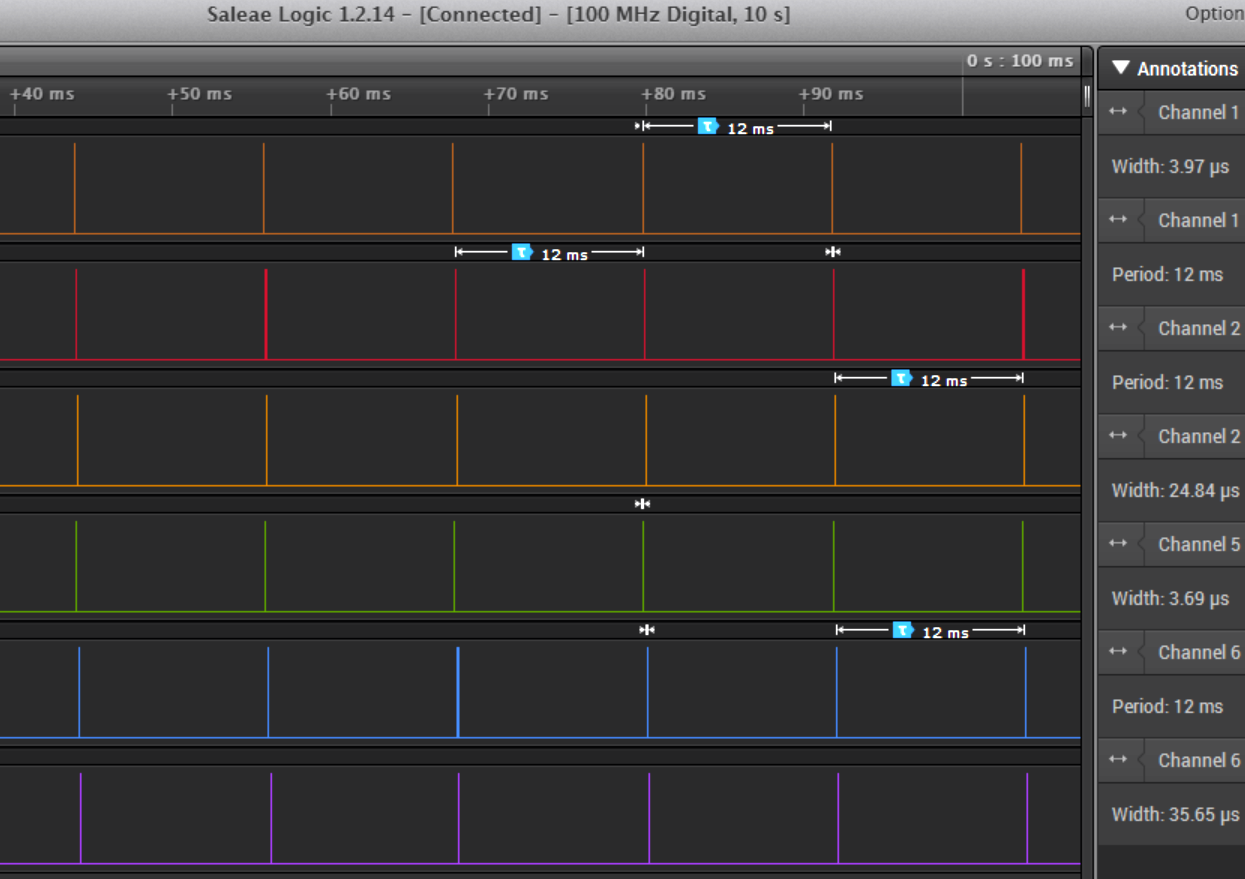

Supplement: Supplementary file 1 [file sensors-18-00628-s001.zip › cpal_codesign_framework/5_introspection_on_target_Raspberry_Pi/01_over_all_2.png]

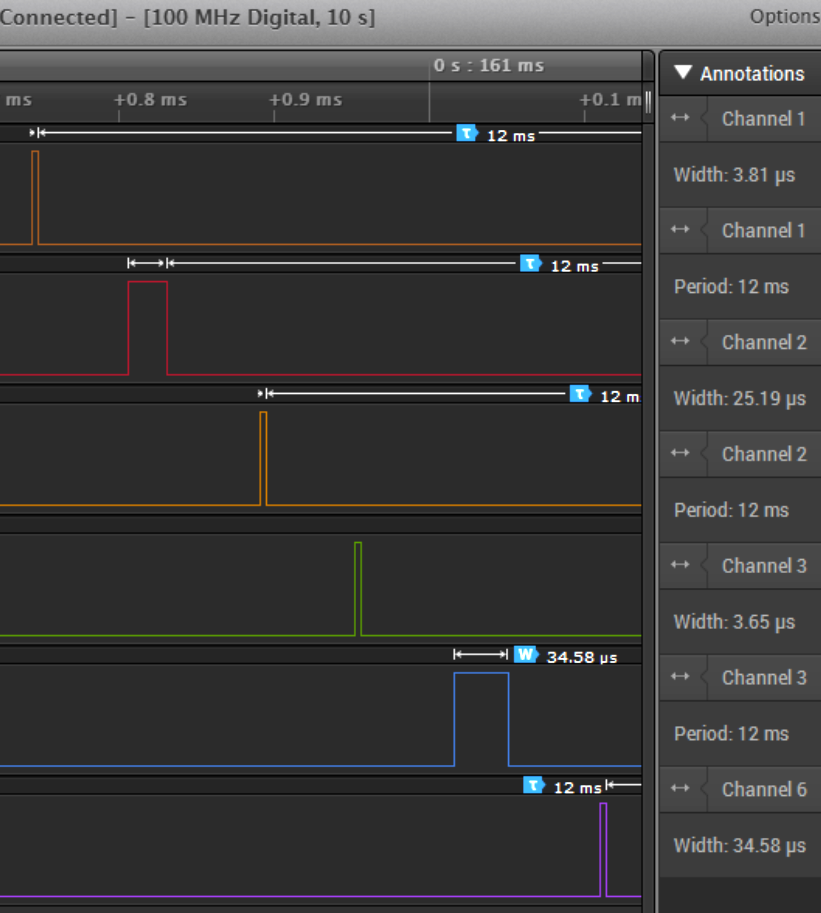

Supplement: Supplementary file 1 [file sensors-18-00628-s001.zip › cpal_codesign_framework/5_introspection_on_target_Raspberry_Pi/02_Zoomed.png]

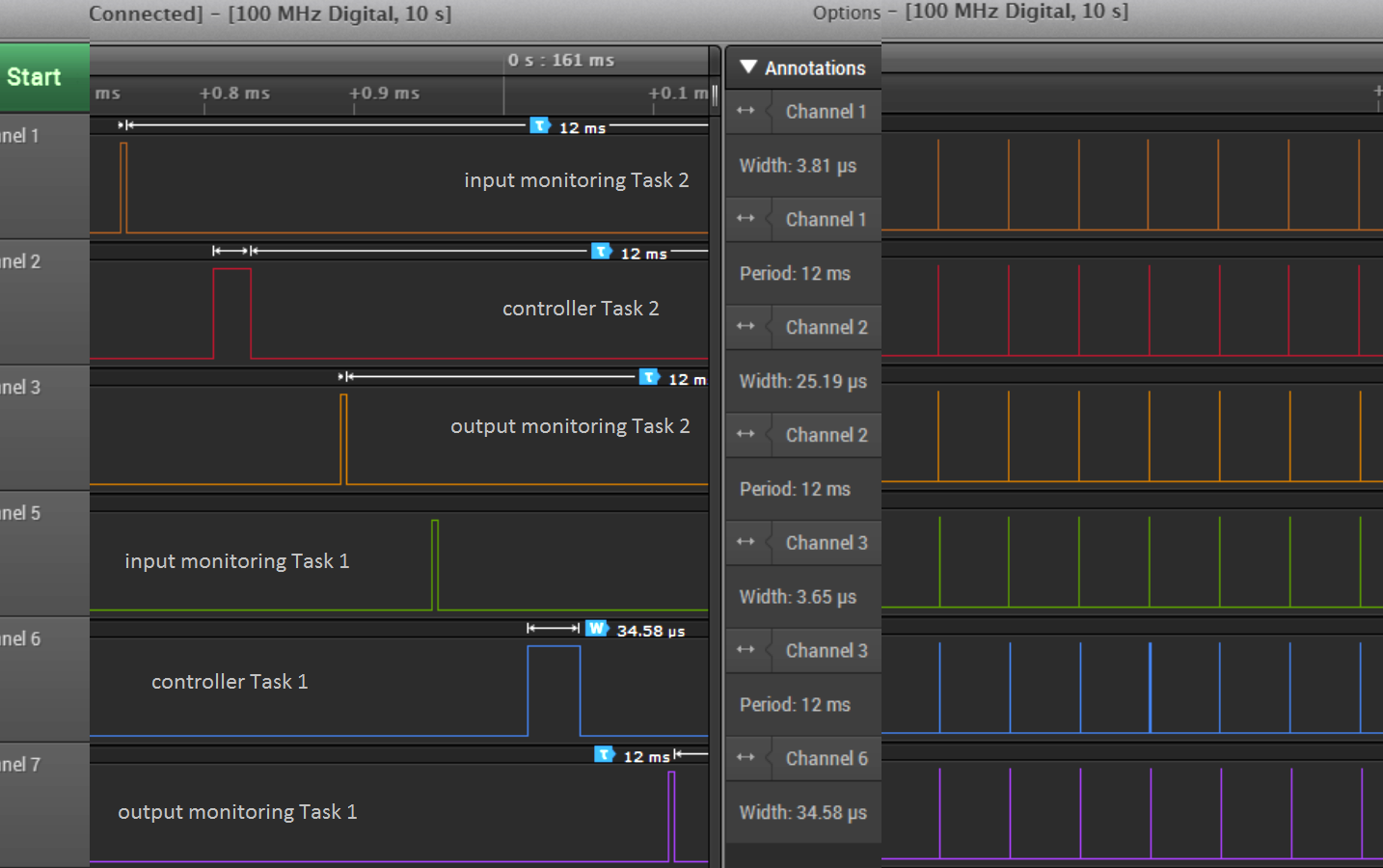

Supplement: Supplementary file 1 [file sensors-18-00628-s001.zip › cpal_codesign_framework/5_introspection_on_target_Raspberry_Pi/03_raspi_mod_target.png]

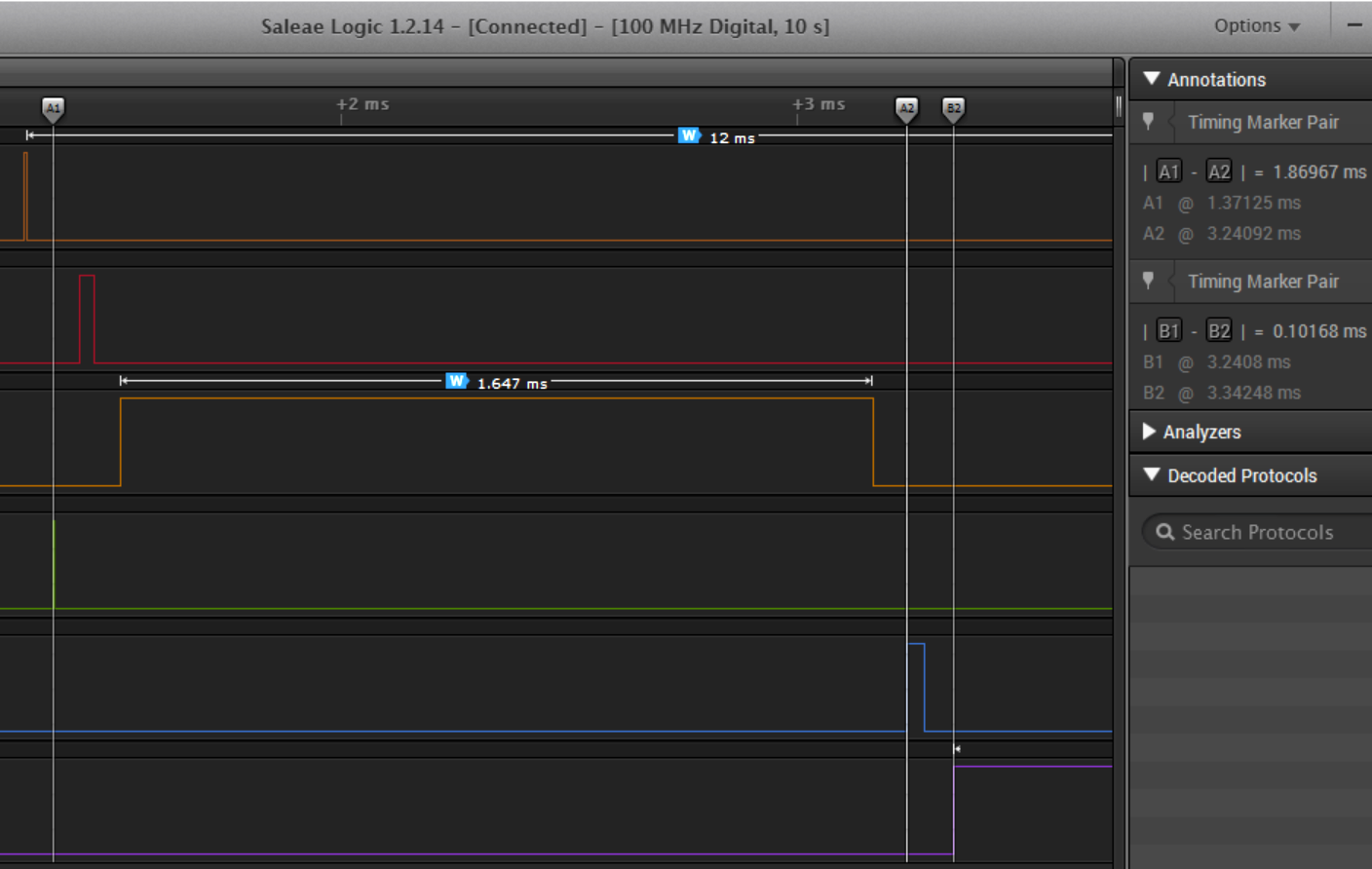

Supplement: Supplementary file 1 [file sensors-18-00628-s001.zip › cpal_codesign_framework/5_introspection_on_target_Raspberry_Pi/04_input_jitter.png]

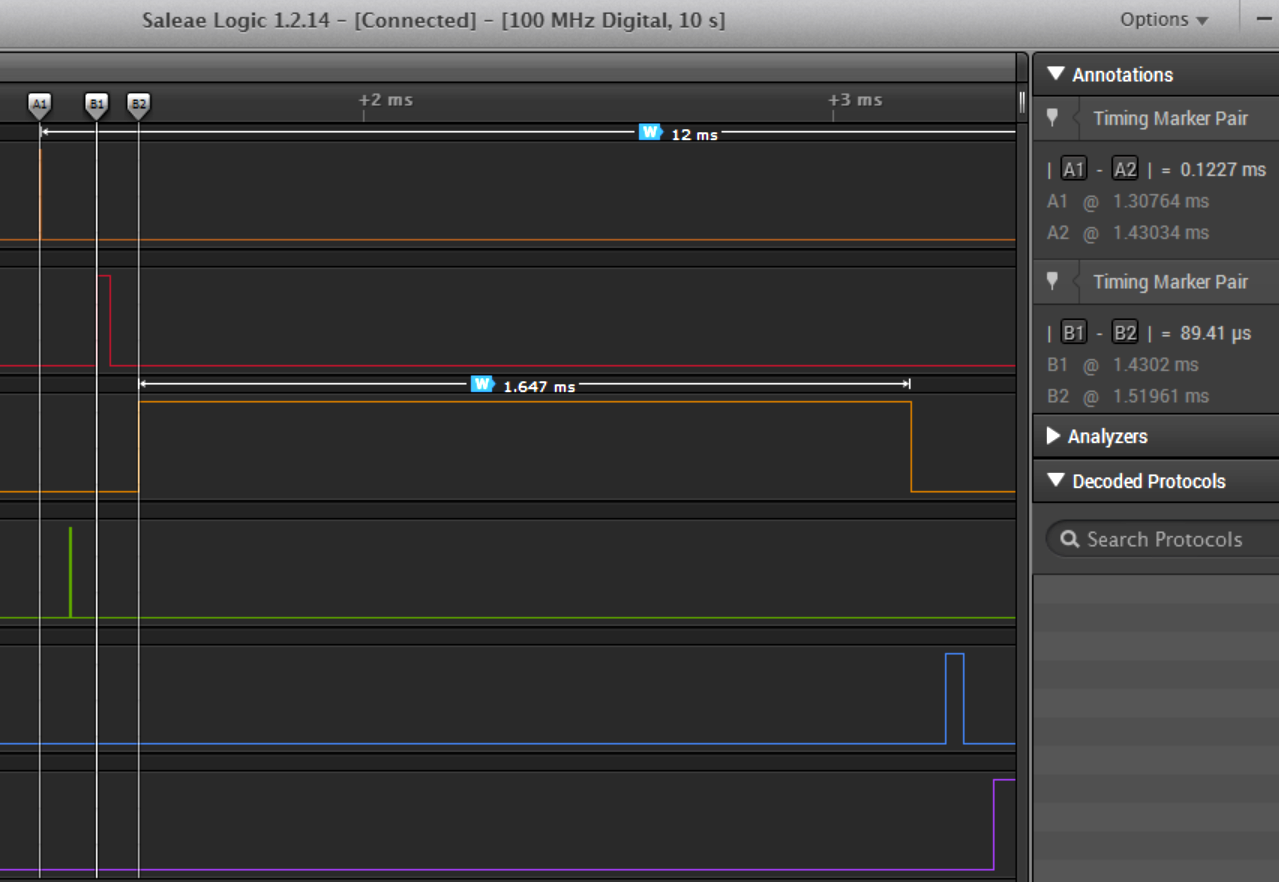

Supplement: Supplementary file 1 [file sensors-18-00628-s001.zip › cpal_codesign_framework/5_introspection_on_target_Raspberry_Pi/04_output_jitter.png]

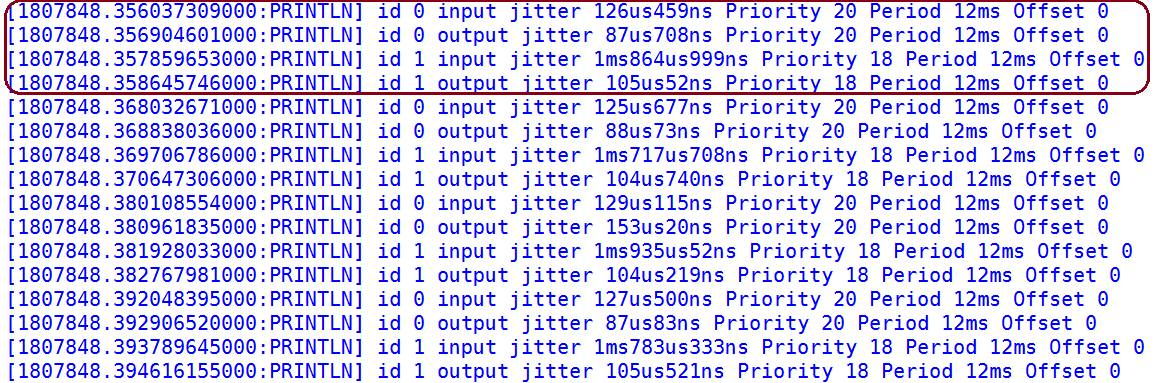

Supplement: Supplementary file 1 [file sensors-18-00628-s001.zip › cpal_codesign_framework/5_introspection_on_target_Raspberry_Pi/04_sample_cli.png]

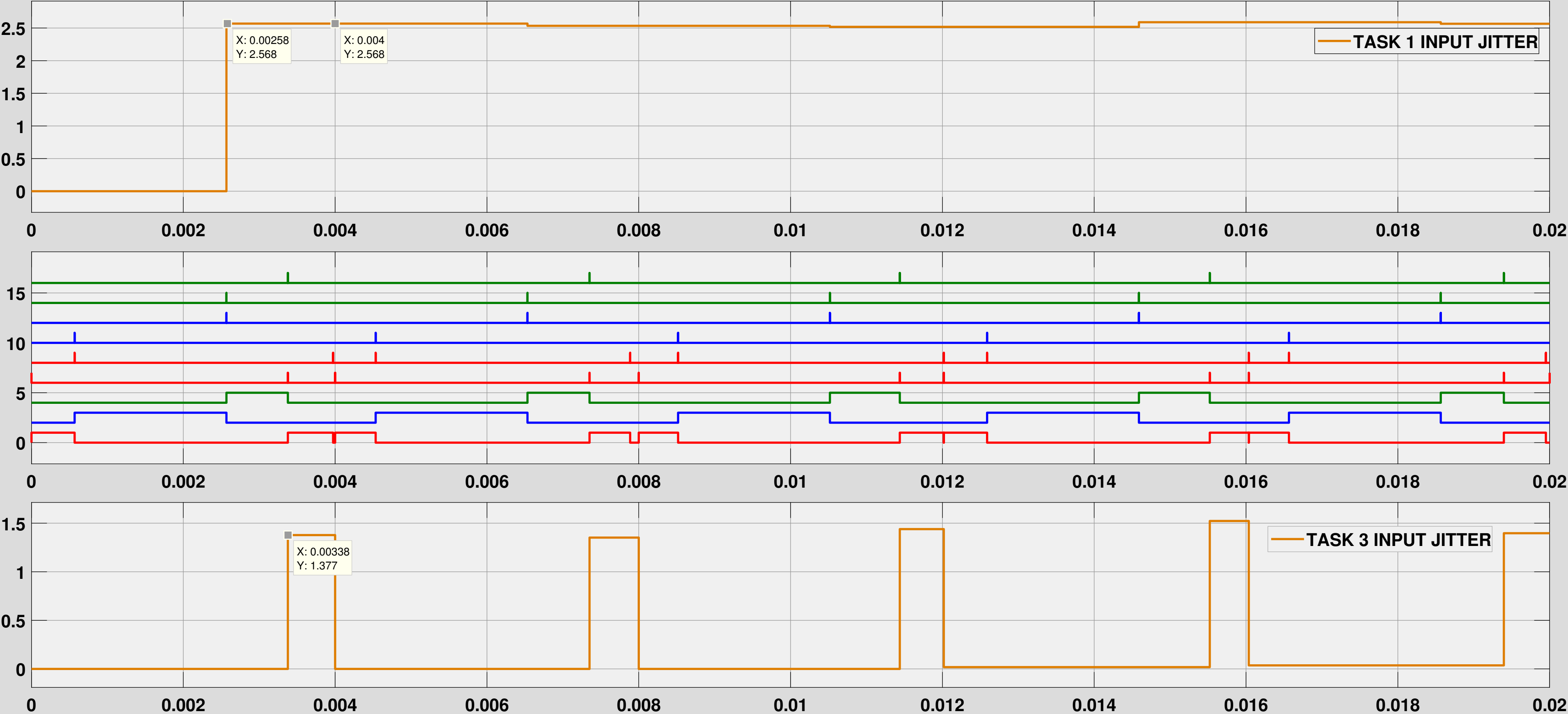

Supplement: Supplementary file 1 [file sensors-18-00628-s001.zip › cpal_codesign_framework/6_introspection_simulation/introspection_graphs/input_jitter_alltask_sample.pdf]

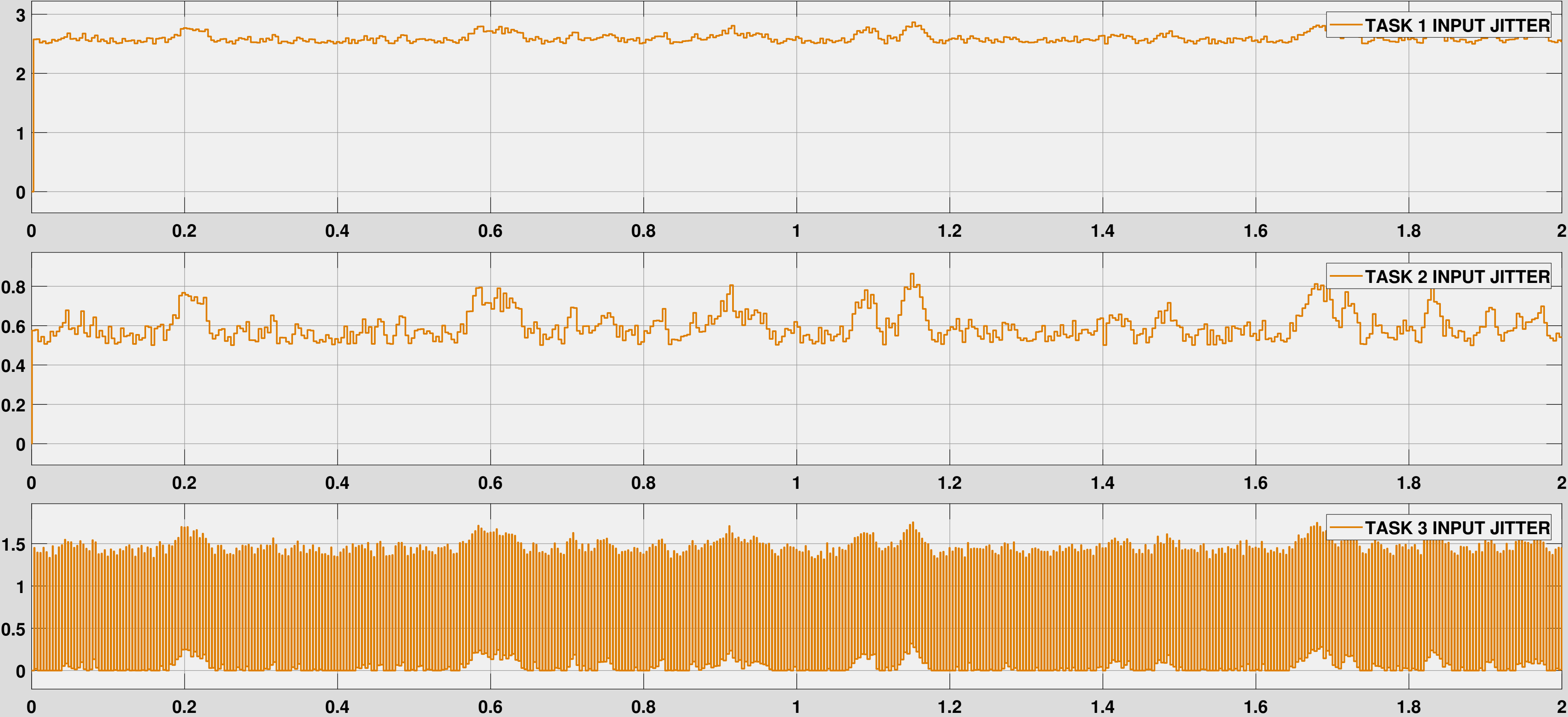

Supplement: Supplementary file 1 [file sensors-18-00628-s001.zip › cpal_codesign_framework/6_introspection_simulation/introspection_graphs/input_jitter_sample.pdf]

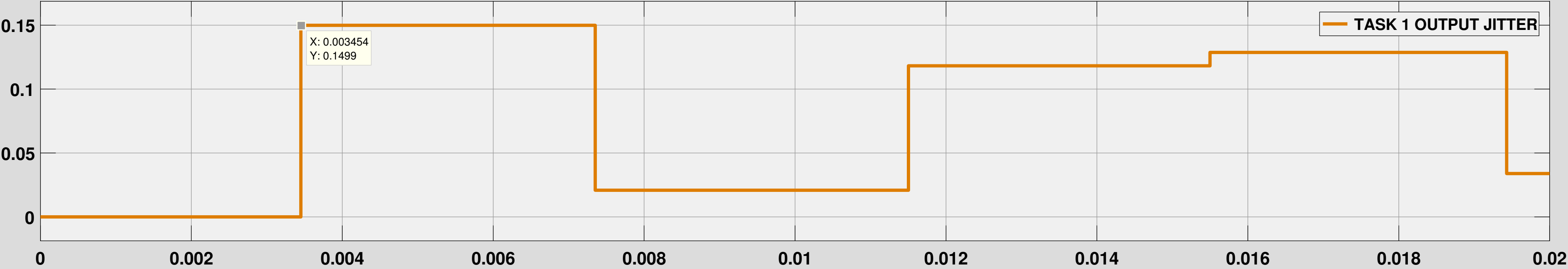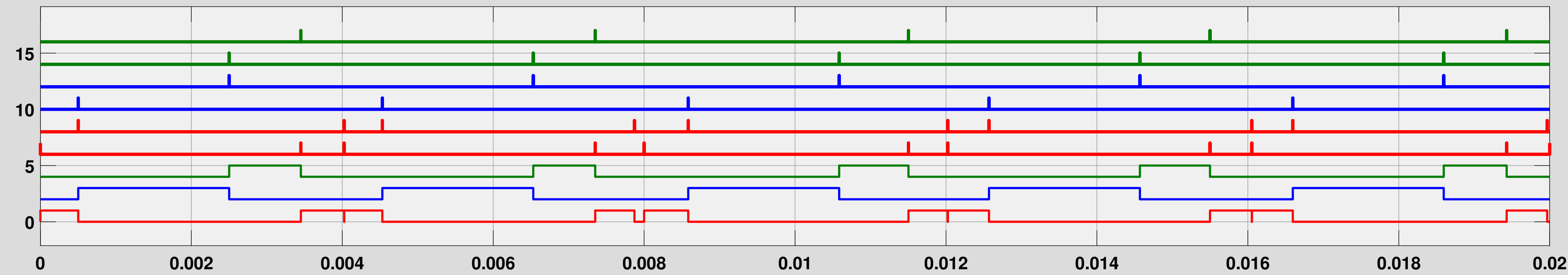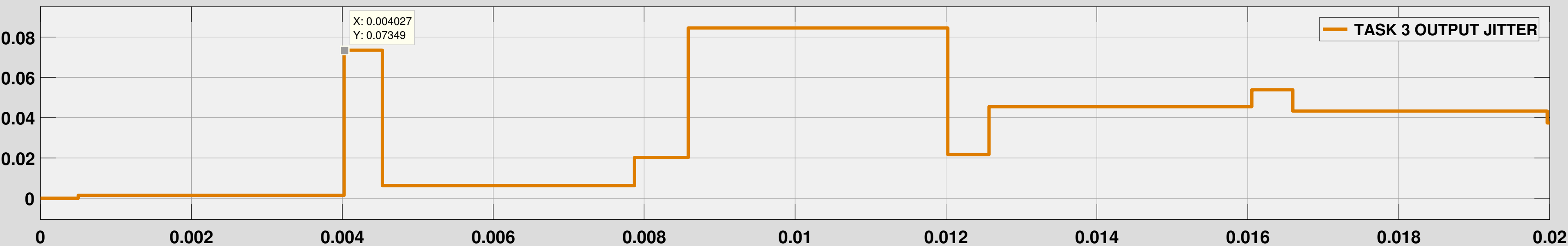

Supplement: Supplementary file 1 [file sensors-18-00628-s001.zip › cpal_codesign_framework/6_introspection_simulation/introspection_graphs/output_jitter_alltask_sample.pdf]

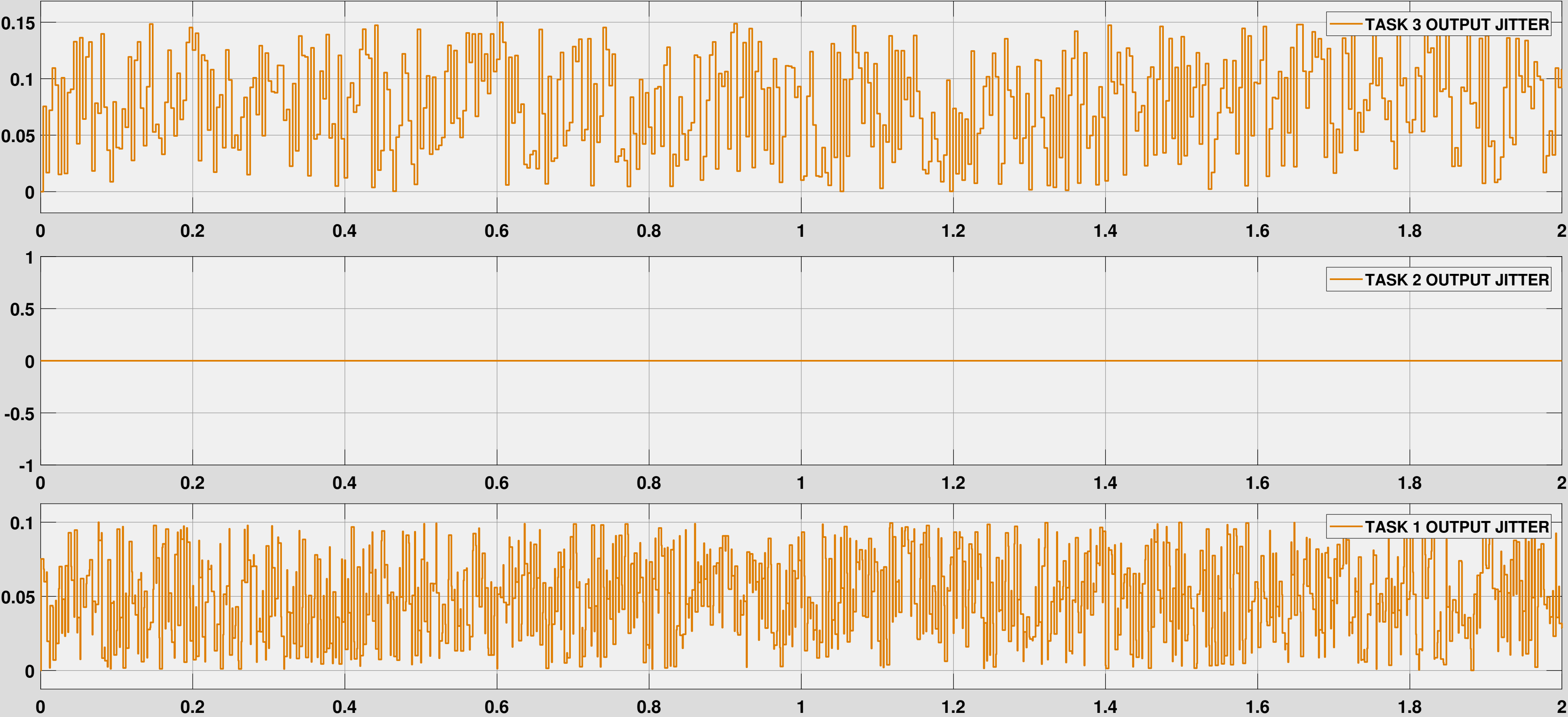

Supplement: Supplementary file 1 [file sensors-18-00628-s001.zip › cpal_codesign_framework/6_introspection_simulation/introspection_graphs/output_jitter_sample.pdf]

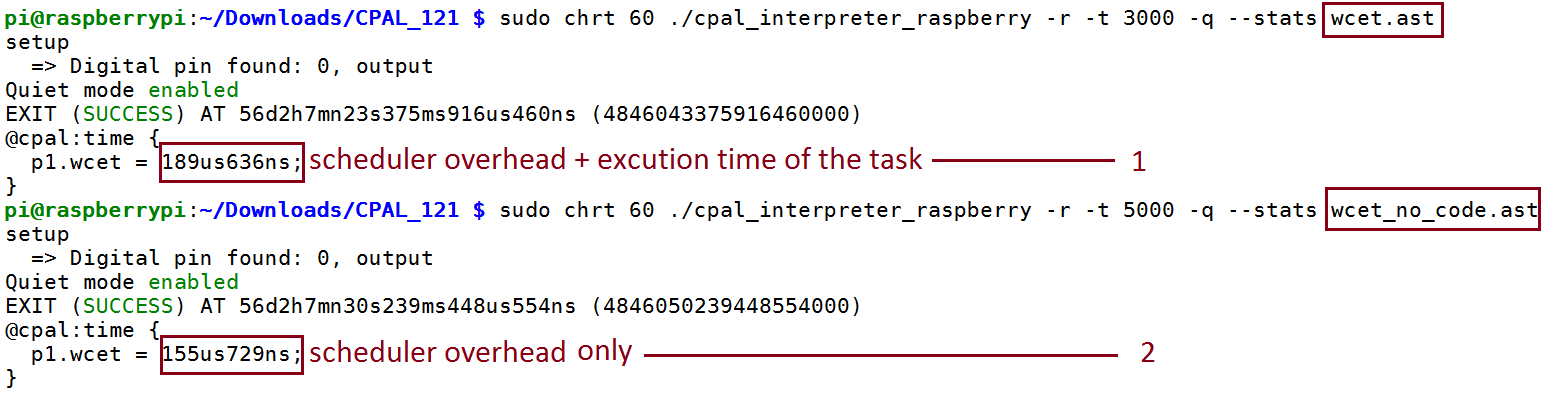

Supplement: Supplementary file 1 [file sensors-18-00628-s001.zip › cpal_codesign_framework/8_wcet/console_clarity.png]

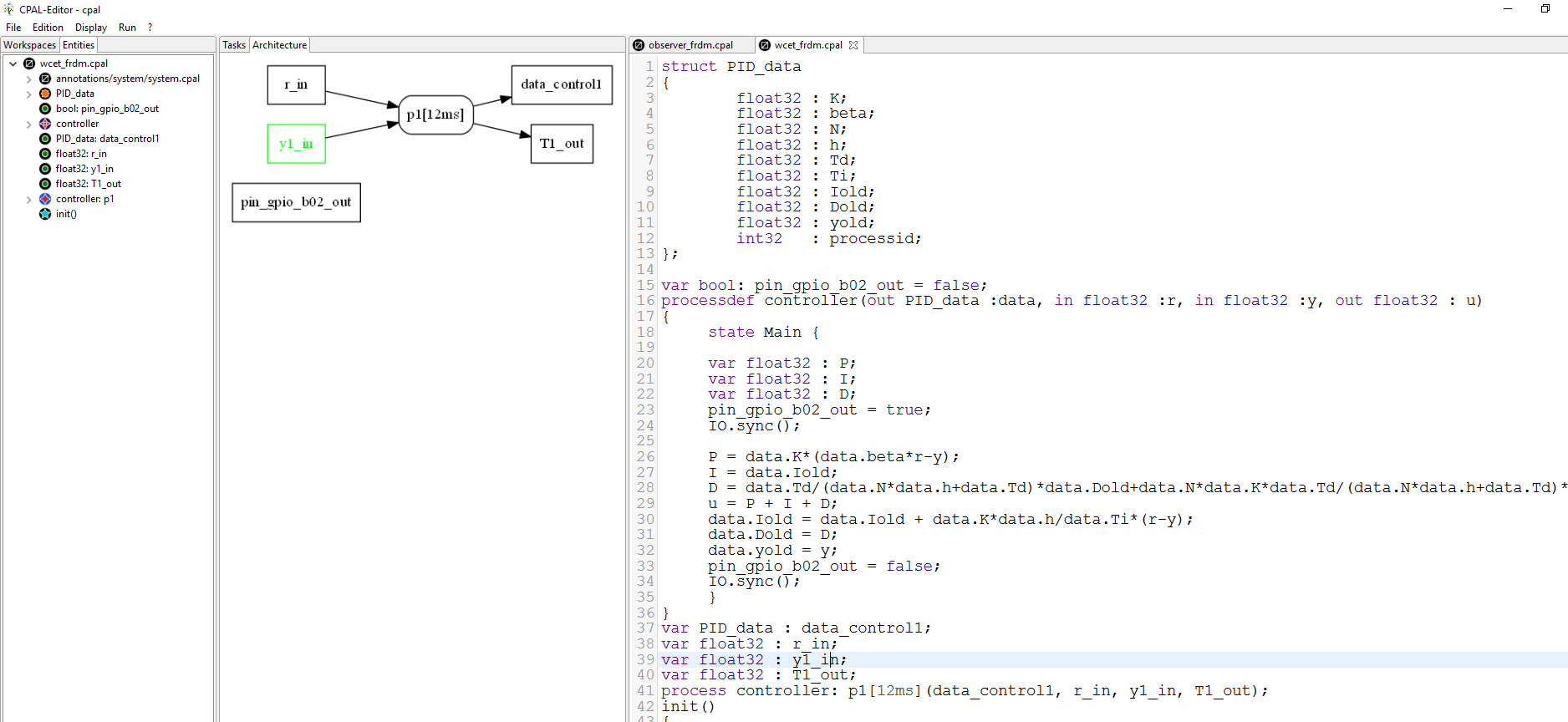

Supplement: Supplementary file 1 [file sensors-18-00628-s001.zip › cpal_codesign_framework/8_wcet/cpal_editor.png]

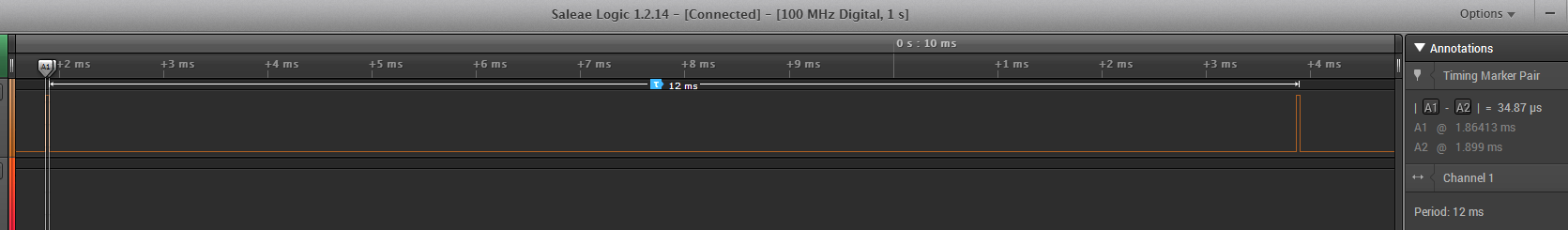

Supplement: Supplementary file 1 [file sensors-18-00628-s001.zip › cpal_codesign_framework/8_wcet/target.png]
